# *Plasmodium falciparum* DNA repair dynamics reveal unique roles for TLS polymerases and PfRad51 in genome diversification

**DOI:** 10.1093/nar/gkaf1275

**Published:** 2025-11-24

**Authors:** Akshay Vishwanatha, Xu Zhang, Yi Jing Liu, Annie Leung, Mikayla Herring, Joseph Visone, Amanda Chan, Susanah Calhoun, Kirk Deitsch, Laura Kirkman

**Affiliations:** Department of Medicine, Weill Cornell Medical College, New York, NY 10065,United States; Department of Microbiology and Immunology, Weill Cornell Medical College, New York, NY 10065, United States; Department of Medicine, Weill Cornell Medical College, New York, NY 10065,United States; Department of Medicine, Weill Cornell Medical College, New York, NY 10065,United States; Department of Medicine, Weill Cornell Medical College, New York, NY 10065,United States; Department of Microbiology and Immunology, Weill Cornell Medical College, New York, NY 10065, United States; Department of Medicine, Weill Cornell Medical College, New York, NY 10065,United States; Department of Microbiology and Immunology, Weill Cornell Medical College, New York, NY 10065, United States; Department of Microbiology and Immunology, Weill Cornell Medical College, New York, NY 10065, United States; Department of Microbiology and Immunology, Weill Cornell Medical College, New York, NY 10065, United States; Department of Medicine, Weill Cornell Medical College, New York, NY 10065,United States; Department of Microbiology and Immunology, Weill Cornell Medical College, New York, NY 10065, United States

## Abstract

The human malaria parasite, *Plasmodium falciparum*, faces unique DNA repair challenges; it is haploid, undergoes asynchronous mitosis termed schizogony, and lacks canonical non-homologous end joining (C-NHEJ). Yet, it has adapted DNA repair pathways that enable survival in distinct environments, including human erythrocytes and hepatocytes, as well as the mosquito vector. *Plasmodium falciparum* chromosomes are partitioned into a conserved core genome and highly diverse subtelomeric regions containing hypervariable, multicopy gene families, including *var*, which encodes a critical parasite virulence factor. The molecular mechanisms maintaining this chromosomal structure remain unclear. Here, we describe specific DNA repair pathways that distinguish hypervariable subtelomeric regions from the conserved core genome. By disrupting the DNA repair enzyme *PfRad51* and TLS polymerases *PfPol*ζ and *PfRev1*, we identified differential irradiation hypersensitivity across the cell cycle for TLSΔ parasites and uniform hypersensitivity for *PfRad51*Δ parasites, highlighting variable roles for these repair pathways. Repair of targeted double-strand breaks demonstrated that PfRad51 is essential for HR-mediated repair in the core genome, whereas a Rad51-independent, homology-directed repair pathway was observed in subtelomeric regions. This previously unidentified alternative repair pathway was independent of TLS polymerases. We propose that these differential DNA repair responses maintain the unique structure that defines *P. falciparum* chromosomes.

## Graphical Abstract

**Graphical Abstract:**
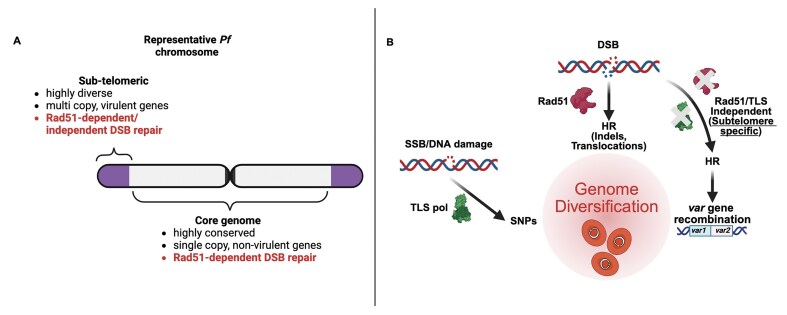

## Introduction

Malaria inflicts a tremendous health and economic burden, with an estimated 263 million malaria cases in 2023 (an increase of 11 million compared to 2022) and a death toll of ~0.5 million globally [1]. The emergence of resistance to artemisinin and partner drugs used in combination therapy to treat malaria poses a significant risk to global efforts to control the disease [1]. Malaria is caused by infection with the unicellular obligate eukaryotic parasite of the genus *Plasmodium*, with *Plasmodium falciparum* being the most virulent of the human malarias. Unique features of the parasite’s genome have been associated with virulence and its propensity to develop drug resistance. *Plasmodium falciparum* has a distinctly skewed genome with 80% AT content, notable repetitive low-complexity regions, and a propensity to accumulate small indels during mitotic division [2]. The parasite has a haploid genome for most of its lifecycle, including all stages in the human host, with a brief diploid stage in the mosquito vector. *Plasmodium falciparum* has 14 chromosomes displaying a high degree of genome plasticity, mostly confined to the subtelomeric regions. These subtelomeric regions can extend to ∼100 kb in length from the chromosome ends and include telomere-associated repeat elements (TAREs) and members of several hypervariable gene families such as *var, rifin* (repetitive interspersed family), *stevor* (subtelomeric variant open reading frame), and *Pfmc-2TM* (*P. falciparum* Maurer’s cleft–2 transmembrane domain protein) [3, 4]. Each family contains multiple genes that exhibit high sequence variation within the genome of a single parasite and between strains isolated from different geographic regions [4–6]. This exceptional genetic variability is essential for the parasite’s ability to avoid the host immune response and thus is a critical component of *P. falciparum* virulence.

Previous reports highlighted the extreme variability of subtelomeric regions relative to the highly conserved core genome [4]; however, the mechanisms by which the parasite maintains this dichotomy remain a mystery. Specifically, it is unclear how parasites partition their genomes to generate the remarkable sequence diversity found within subtelomeric regions while simultaneously maintaining a highly conserved core genome. It is likely that DNA repair mechanisms play an important role in genetic diversification, particularly of the dominant variant surface antigen, erythrocytic membrane protein 1 (PfEMP1), encoded by the multicopy *var* gene family, which is clustered in subtelomeric and several internal chromosomal regions (Supplementary Fig. S1) [3]. Mechanisms including non-allelic homologous recombination (NAHR), alternative end joining (Alt-EJ), gene conversion (GC), and telomere healing (TH) have been proposed to contribute to this diversity during the asexual erythrocytic stages [7–10], although the targeting of these mechanisms specifically to the variable regions of the genome is not understood. Therefore, understanding how parasites employ these different DNA repair pathways will provide insight into the complex process of genome diversification.

As a haploid organism lacking non-homologous end-joining (NHEJ), *P. falciparum* possesses a unique genome maintenance system crucial for survival within the host. DNA repair mechanisms are required to overcome DNA damage induced by genotoxic stress, external agents such as mutagens, ionizing radiation, and various antimalarials [11–14]. As the parasite proceeds through the intraerythrocytic development cycle (IDC), it undergoes schizogony, during which one parasite replicates asynchronously to form ∼30 daughter cells. This requires multiple rounds of DNA replication; thus, the parasite undergoes significant replication stress. Damaged bases or mistakes during DNA replication result in replication fork stalling, and DNA damage must be repaired for replication to proceed. In addition to various repair mechanisms to remove damaged or mismatched bases, cells have developed specific processes that tolerate mistakes and allow replication to proceed. These processes are called replicative DNA damage bypass pathways or translesion synthesis (TLS) [15]. TLS is a highly conserved process mediated by specialized polymerases that replicate opposite and past damaged bases with lower fidelity, called TLS polymerases. The lower fidelity of these polymerases carries an increased risk of mutagenesis, which can be detrimental but also serve as a source of genomic diversification [15, 16]. We recently reported the potential importance of TLS components in diversifying multigene families in *P. falciparum* through ectopic recombination [17]. Rodent malaria parasites also harbor multicopy gene families; however, there is a high degree of sequence identity between different isolates [18], which contrasts with much higher diversity between multicopy gene families in *P. falciparum* isolates. As rodent parasites also lack the machinery for TLS, we hypothesized that this could account for the relative lack of multicopy gene diversification and that TLS polymerases may play an active role in multicopy gene diversification in *P. falciparum*, potentially in concert with mediators of HR such as PfRad51 [17]. Components of the TLS complex identified *in silico* in *P. falciparum* include the scaffold protein subunit *PfRev1* and the catalytic subunit *PfPol*ζ/*Rev3*. No Rev7 homolog has been identified, though there are two HORMA domain-containing proteins that could potentially serve as Rev7 orthologs, though neither has been studied to date.

Another lethal type of DNA damage is double-strand breaks (DSBs), which are repaired mainly by the NHEJ and the homologous recombination (HR) repair pathways. *Plasmodium falciparum* and all closely related blood-borne apicomplexan parasites lack the components of canonical NHEJ, making HR the major pathway for repairing DSBs [19–21]. Since *Plasmodium* is haploid throughout its IDC and lacks homologous pairs of chromosomes, it is surprising that it depends on HR for DSB repair. A relatively inefficient alt-EJ pathway was identified that can act to repair DSBs in the absence of homologous templates [20, 22]. In addition, we and others have reported how antigen diversification occurs through mitotic recombination mediated by HR in *P. falciparum* [7–10, 23, 24]. A defining feature of HR is the utilization of homologous DNA sequences as a repair template, where the broken DNA molecule finds and invades the homologous DNA molecule. The Rad51 recombinase, assembled on the single-stranded DNA at the site of the break as an oligomeric nucleoprotein filament, performs this essential function [25].

Based on data suggesting the importance of the TLS polymerases and the HR pathway in the maintenance and diversification of the parasite genome, we set out to determine the general function of these proteins in *P. falciparum* as well as their specific role in genome diversification.

## Materials and methods

### Culture and genetic manipulation of parasites

Parasites were cultured in a standard culture system at 5% hematocrit in media containing RPMI 1640 (Corning Life Sciences, Tewksbury, MA, USA), 0.5% Albumax II (Invitrogen, Carlsbad, CA, USA), 0.25% sodium bicarbonate, and 0.1 mg/ml gentamicin in an atmosphere of 5% oxygen, 5% carbon dioxide, and 90% nitrogen. Culture media was supplemented with the required selections [WR99210 (Jacobus Pharmaceuticals, Plainsboro Township, NJ; 2.5 nM final concentration), Blasticidin (ThermoFisher, Fair Lawn, NJ; 2 μg/ml final concentration), or DSM1 (Sigma–Aldrich, St. Louis, MO; 1.6 μM final concentration)] as per the resistance markers present in our transgenic cell lines. Resistance cassettes used include human dihydro folate reductase (hDHFR), Blasticidin S deaminase (BSD), and Dihydroorotate dehydrogenase (DHODH). Clonal parasite lines were obtained by limiting dilution [26]. Parasites were transfected by electroporation [27] using derivatives of the plasmids p*L6_eGFP* and p*UF1_Cas9* for CRISPR/Cas9-based genome editing as described by Ghorbal and colleagues [28]. Homology blocks for genome modification were amplified from parasite genomic DNA by polymerase chain reaction (PCR), single guide sequences were synthesized as oligos, annealed, and both were inserted into p*L6* by infusion cloning (Clontech, Takara Bio USA, Mountain View, CA, USA). The plasmids generated are listed in Supplementary Table S1. Single guide sequences and sequences used as homology blocks for CRISPR modifications are listed in Supplementary Tables S2 and S3. Growth of the parasites was monitored using flow cytometry as described [29, 30]. For normal growth conditions, parasites were diluted once they reached 1%–2% parasitemia, and the dilution factor was recorded to calculate cumulative parasitemia. For irradiated cultures, no dilutions were made, and growth was tracked in real time. The parasite cell lines used in this study are listed in Supplementary Table S4.

### RNA isolation, cDNA synthesis, and qPCR

Parasites were synchronized using 5% sorbitol treatment for two rounds and 36 h later late stages were isolated. Synchronization was verified by microscopy and flow cytometry. RNA was extracted with TRIzol (Invitrogen, Carlsbad, CA, USA) and purified on PureLink (Invitrogen, Carlsbad, CA, USA) columns following the manufacturer’s protocols. Isolated RNA was treated with Deoxyribonuclease I (DNase I) (Invitrogen, Carlsbad, CA, USA) to degrade contaminating genomic DNA. Complementary DNA (cDNA) was synthesized from ~1000 ng of RNA in a reaction that included Superscript II RNase H reverse transcriptase (Invitrogen, Carlsbad, CA, USA) as described by the manufacturer. Control reactions in the absence of reverse transcriptase were employed to verify a lack of gDNA contamination. Quantitative PCR was performed using a Quant Studio 6 Flex 489 real-time PCR machine (Thermo Fisher, Fair Lawn, NJ, USA) using iTaq Sybr Green (Bio-Rad, Philadelphia, PA, USA). Quantities were normalized to seryl-tRNA synthetase (PF3D7_0717700). ΔCT for each individual primer pair was determined by subtracting the individual CT value from the CT value of the control and converting to relative copy numbers with the formula 2^ΔCT^. All quantitative real-time reverse transcription polymerase chain reaction (qRT-PCR) assays were performed in a 384-well format PCR machine enabling duplicate or triplicate runs performed simultaneously. Biological replicates were prepared from independent RNA extractions. Transcriptional profiles of individual genes are presented as bar graphs and standard deviations from biological replicates are shown with error bars.

### Genomic DNA extraction

Infected red blood cells from a 10-ml culture (∼4%–5% late stages) were centrifuged at 2000 × *g*. The supernatant was discarded, and the pellet was resuspended in 1 ml of phosphate-buffered saline and 15 μl of 10% saponin. The parasite pellets were spun at 13 500 × *g* for 3 min and washed twice in 1 ml of PBS. The pellet was then taken up in 400 μl Tris–sodium chloride–ethylenediaminetetraacetic acid buffer, along with 80 μl of 10% sodium dodecyl sulfate and 40 μl of 6 M NaClO_4_. This suspension was placed on a rocker at room temperature overnight. The following morning, an equal volume of phenol-chloroform-isoamyl alcohol (25:24:1) was added and gently vortexed. We did not vortex samples slated for Nanopore sequencing to maintain the length of the DNA and instead gently mixed them by inverting the tube 5–10 times. Samples were then centrifuged at 16 000 × *g* for 5 min. The final aqueous phase was ethanol precipitated and resuspended in 50 μl of sterile distilled H_2_O. The final DNA concentration was determined by absorbance at 260 nm using a NanoDrop machine.

### Sequencing of parasite genomic DNA

Sequencing libraries were prepared from 1 μg of unsheared gDNA (as measured using the Qubit dsDNA BR assay [Life Technologies, Carlsbad, CA, USA]) using the Oxford Nanopore Technologies ligation sequencing kit version 1D LSK 108 (Oxford, UK). This approach minimizes the potential shearing of DNA template fragments and entirely avoids PCR artifacts. A manufacturer-supplied adapter nucleoprotein complex is ligated to native gDNA to facilitate the loading of long library molecules into the protein nanopores. Nanopore sequencing was conducted with an Oxford Nanopore Technologies MinIon instrument using MIN-106/R9.4.1 flowcells. All samples were sequenced using a standard 48 h run controlled by Oxford Nanopore Technologies MinKnow software, and raw data were basecalled with Albacore version 2.2.4. FASTQ files were extracted from basecalled FAST5 files with Poretools [31].

Illumina whole genome sequencing was performed using NovaSeq 6000 system to get ~5 million reads per sample. Sample library was prepared using Kapa Hyper library prep kit (Roche, Wilmington, MA, USA) and run on an S2 flow cell with 2 × 100 cycles to obtain paired-end reads in the fastq format.

All the sequencing data can be accessed at https://www.ncbi.nlm.nih.gov/bioproject/PRJNA1244413.

### Irradiation of parasites

Irradiation of the parasites was performed using RS 2000 Biological Research X-ray Irradiator (Rad Source Technologies, Buford, GA, USA). Briefly, tightly synchronized (∼98%; either ring or late stage) parasite cultures were obtained using repeated 5% sorbitol or Percoll/sorbitol treatment to eliminate later-stage parasites [32, 33]. Parasites were exposed to the required amount of irradiation utilizing the level 3 shelf, which supplied ∼2.1 Gy/min. The culture was maintained by media change and by replenishing blood until growth was noted in the irradiated and control parasite cultures. Once growth was re-established, the parasites were irradiated again for the second time and then a third time. After the third round of irradiation, the gDNA from the surviving parasites was isolated as mentioned above and purified using Zymo Research genomic DNA clean and concentrator kit (Zymo Research, Irvine, CA, USA).

### Quantification of SNPs, indels, and translocations

To quantify SNPs and Indels from the Illumina sequencing datasets, reads were trimmed using Trimmomatic [34] to remove adapters, read quality was checked using FastQC [35], and the genome was indexed and aligned using BWA-MEM [36]. Duplicates were marked using sambamba [37] and the bam file was sorted and indexed using samtools [38]. Variants were called using FreeBayes [39] and the VCFtools package was used to filter out variants having quality (QUAL) scores <30 and DP scores <10. To determine the translocations from Nanopore reads, bam files from the raw fastq files were generated using minimap2 [40], sorted and indexed using samtools [38], and variants called using sniffles [41]. SNPs, indels, and translocations unique to the irradiated cell lines were identified by normalizing to the respective non-irradiated parental cell line using the vcf-isec command of vcftools [42] and were plotted into circos plots using the R package circlize [43]. We used SNPeff tool to quantify, annotate, and analyze the functional effects of the SNPs and Indels [44]. Translocations identified by sniffles were visually confirmed using IGV [45] before circos plotting. All the codes for the analysis performed can be accessed at Zenodo here: https://doi.org/10.5281/zenodo.15312139.

### Local genome assembly

To assemble the genome at specific location (*var2csa* target region for DNA repair studies), bam files from Nanopore sequencing were filtered to obtain a localized assembly for the desired region using Flye assembler [46]. The assembled genomic region was then annotated manually to identify the sequence similarity with donor plasmids to confirm homologous recombination events. All the codes for the analysis performed can be accessed at Zenodo here: https://doi.org/10.5281/zenodo.15312139.

## Results

### Creation of homologous recombination and translesion synthesis knockout mutants

**Figure 1.**
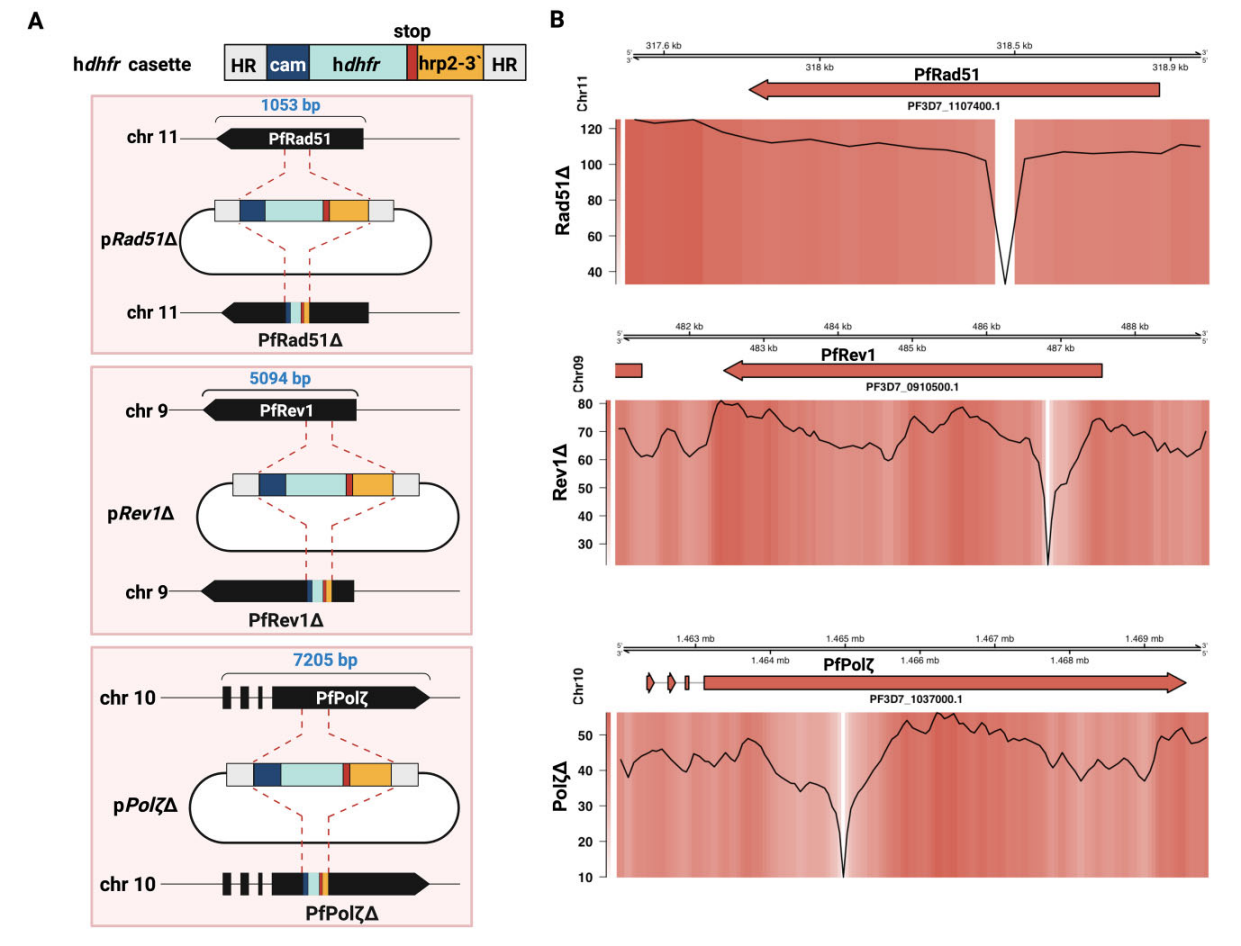
Creation and confirmation of disrupted *PfRad51, PfRev1*, and *PfPol*ζ. (**A**) Overview of the molecular approach used for creating gene disruptions of *PfRad51, PfRev1*, and *PfPol*ζ. The top cartoon shows the hDHFR (dihydrofolate reductase) cassette used for plasmid selection and integration. (**B**) Coverage plots of Nanopore sequencing results of the *PfRad51*Δ, *PfRev1*Δ, and *PfPol*ζΔ. The top panel denotes the genome region, chromosome, and gene. The bottom panel has both heatmap and a line plot to show the integration site where the hDHFR cassette has been integrated, which can be seen as a drastic drop in the coverage (large dip in the line plot coinciding with the white region in the coverage plot).

To study the function of *PfRad51* (PF3D7_1107400) and TLS polymerases *PfRev1* (PF3D7_0910500) and *PfPol*ζ (PF3D7_1037000) in *P. falciparum*, we first tested if these genes were essential for parasite viability. Rad51 is especially interesting because it is essential for survival in vertebrates, where a null mutation leads to embryonic lethality and death of mammalian cell lines [47], whereas Rad51 is not essential in other unicellular organisms such as budding yeast [48], *T. brucei* [49], fission yeast *S. pombe* [50], and *S. japonicus* [51]. First, we generated a domain knockout of *PfRad51* using CRISPR/Cas9 (Clustered Regularly Interspaced Short Palindromic Repeats/CRISPR-associated protein-9 nuclease). We made targeted domain knockouts, which led to gene disruption and a frame shift in the coding region [52–60]. As shown in Fig. 1A (top cartoon), a repair block containing the selection marker hDHFR, providing resistance to the drug WR99210 (WR) with flanking homologous regions upstream and downstream of the selected PAM (protospacer adjacent motif) site in the target gene, *PfRad51*, constituted the donor plasmid [28]. The donor plasmid also encoded a single guide RNA targeting the sequence coding for the ATP binding domain (Walker A motif) (Supplementary Fig. S2A) of the protein (TTATTTGGTGAATTTCGTAC|AGG). A similar strategy was used to generate domain knockouts of the TLS polymerases *PfRev1* (ATATTAATAGTTCAAAACGG|AGG, BRCT domain) and *PfPol*ζ (TTATCTTTTTAATGAAAGTG|AGG, upstream of DNA-directed DNA polB domain) using CRISPR/Cas9 as outlined in Fig. 1A and Supplementary Fig. S2A. Rev1 is not essential for the survival of higher eukaryotes, while Polζ is essential during development in mammalian systems [25–62], and both genes are non-essential in yeast [63]. The generated donor plasmid (Supplementary Table S1), along with a Cas9 plasmid (p*UF1-Cas9*) [28], was co-transfected into a wild-type (WT) 3D7 background. Clonal lines were generated representing the three domain knockout mutants: *PfRad51*Δ, *PfRev1*Δ, and *PfPol*ζΔ. Gene disruptions were confirmed by Illumina and Nanopore whole genome sequencing, as shown in Fig. 1B. The loss of part of the gene and replacement by the plasmid selection cassette is represented by the sudden loss of coverage (Fig. 1B). In addition, RT-qPCR of *PfRad51*Δ, *PfRev1*Δ, and *PfPol*ζΔ confirmed that our gene modifications reduced transcripts of the target genes to near undetectable levels (Supplementary Fig S2A and B). The successful transfections and comparable growth rates of the mutant lines to the WT 3D7 line indicate that in *P. falciparum*, these three genes are not essential for survival under normal growth conditions (Supplementary Fig. S2C). These parasites with domain disruptions are termed knockouts in the rest of the manuscript.

### *Plasmodium falciparum* TLS polymerases are essential for the survival of irradiated ring-stage parasites

**Figure 2.**
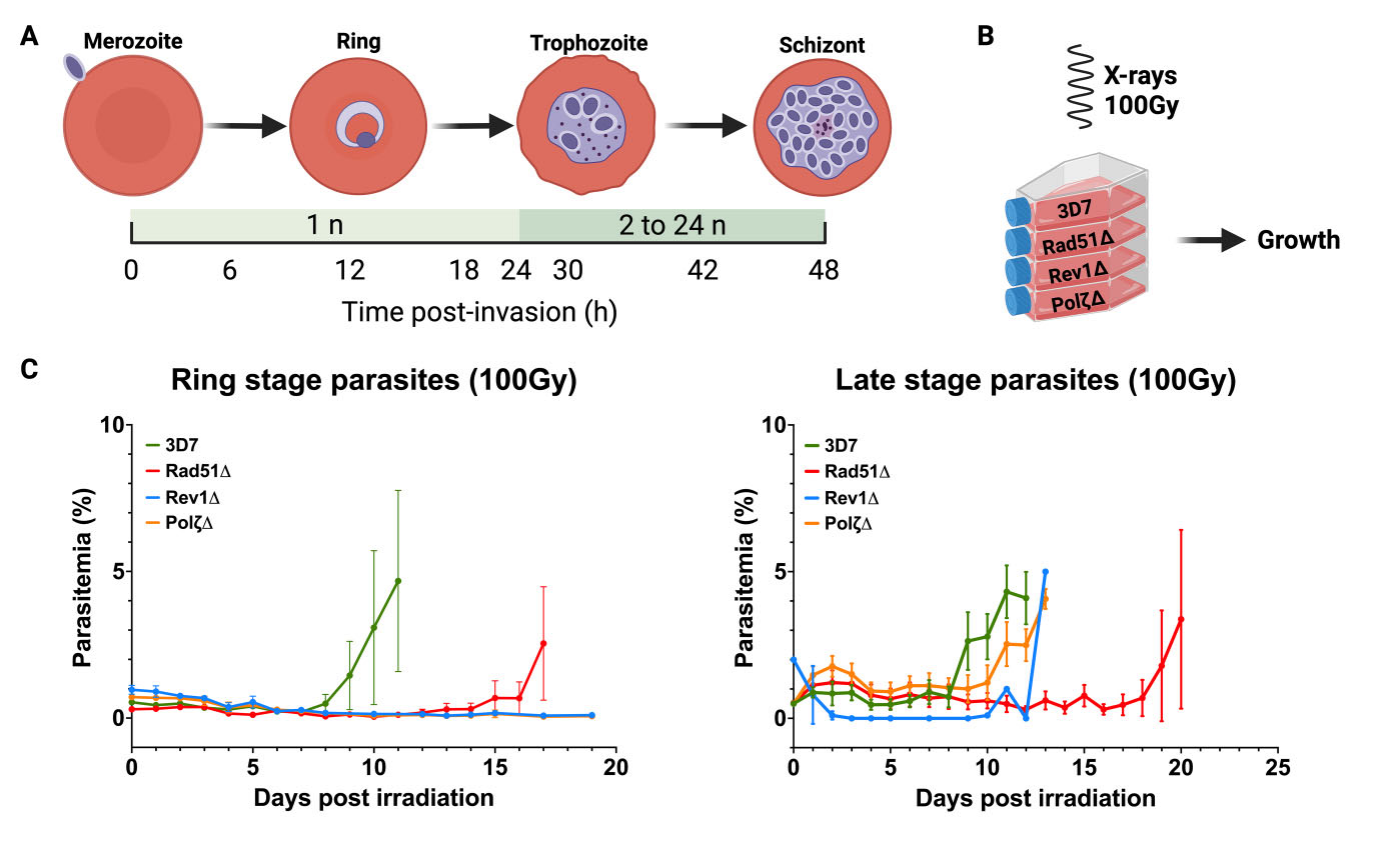
Growth analysis of knockout cell lines. (**A**) The top panel represents the main stages of the intraerythrocytic developmental cycle (IDC) of *P. falciparum*. The bottom panel shows the change in DNA content over the different stages of the IDC [IDC tracked as hours post-invasion (hpi)]. (**B**) Overview of the irradiation exposure of the four different cell lines: WT, *PfRad51*Δ, *PfRev1*Δ, and *PfPol*ζΔ. (**C**) Growth curve of 100 Gy irradiated ring stage (0–24 hpi) and late stage (24–48 hpi) parasites expressed as percent parasitemia (number of infected cells/total red blood cells). The error bars represent SD (*n* = 3). Growth of non-irradiated parasites from WT 3D7 and mutant cell lines showed normal growth under standard growth conditions (Supplementary Fig. S2).

The IDC of *P. falciparum* begins with the invasion of a red blood cell (RBC) by a single merozoite containing one copy of each chromosome. This merozoite develops into the ring form from 0 h to 24 h post-invasion and maintains a single copy of the genome. From 24 to 48 h post-invasion, the parasites multiply via schizogony, marked by multiple rounds of DNA replication and asynchronous nuclear division, yielding 16–32 daughter cells. As the IDC progresses and DNA replication is ongoing, there is an opportunity for replicated DNA to serve as a template for HR to repair DSBs (Fig. 2A) [64–67]. Given this difference, it is reasonable to propose that different pathways are likely responsible for DNA repair across parasite developmental stages. For example, the lack of a template for repair in ring-stage parasites and the absence of NHEJ would suggest a greater reliance on alternate repair pathways. Consistent with this idea, sensitivity to DNA damage also varies depending on the parasite’s position within the replicative cycle [10, 68]. Therefore, to further investigate this hypothesis, the parasites with disrupted *PfRad51* and the TLS polymerases described in the previous section were tested for their sensitivity to DNA damage at different points in the replicative cycle using X-rays as a source of ionizing radiation (Fig. 2C). Ionizing radiation, such as X-rays, is known to induce multiple kinds of DNA damage, including base damage, single-strand breaks (SSB), and DSBs. Each requires a different repair pathway for the exposed cell to recover, such as nucleotide excision repair (NER), base excision repair (BER), mismatch repair (MMR), and DSB repair pathways [69].

*PfRad51*Δ, *PfRev1*Δ, and *PfPol*ζΔ, as well as WT 3D7 parasites, were exposed to 100 Gy irradiation at ring- and late-stages of the replicative cycle. Our previous work indicated that at this irradiation dose, virtually all parasites sustain damage, and those parasites that survive display longer telomeres, a hallmark of recovery from DNA damage [70]. Cultures were then monitored for up to three weeks to determine how the loss of different DNA repair proteins affected the parasites’ ability to repair DNA damage and reestablish growth. Non-irradiated cultures were included as controls in each experiment, and as expected, without DNA damage, these cultures reached a max parasitemia for *in vitro* growth without dilution after two IDC cycles (Supplementary Fig. S2D). For both ring- and late-stages, WT 3D7 parasites recovered from an initial growth inhibition within nine days of irradiation exposure (Fig. 2C, green line), thereby establishing the time required for recovery when all DNA repair pathways are active. Given that malaria parasites are thought to depend almost exclusively on HR to repair DSBs, and since Rad51 is required for HR in model organisms, we anticipated a near-complete elimination of recovery in the *PfRad51*Δ line. We also predicted that the loss of Rad51 might cause a more pronounced effect in late-stage parasites, when HR would further dominate DSB repair. Remarkably, both ring- and late-stage parasites were able to recover from irradiation in the absence of Rad51 (15 days for ring-stage parasites versus 19 days for late stages), and the loss of *PfRad51* did not appear to be associated with a substantial difference in survival between these two stages (Fig. 2C, red line). These data indicate that, despite the presumed dependence on HR for DSB repair in malaria parasites, sufficient alternative repair activity exists to enable reproducible recovery from extensive DNA damage in the absence of Rad51.

We then examined the effect of the loss of TLS polymerases on parasite recovery from the same irradiation dose. In stark contrast to our findings in the *PfRad51*Δ line, we observed that both *PfRev1* and *PfPol*ζ are essential for the survival of irradiated ring-stage parasites (Fig. 2C, blue and orange lines), as the knockouts fail to recover after >20 days. Interestingly, the loss of TLS polymerases had little effect on the parasites’ ability to recover from irradiation in later stages, when *PfRad51*-mediated HR presumably dominates (Fig. 2C, blue and orange lines). The essentiality of the TLS polymerases specifically during the ring stage suggests TLS is critical to repair DNA damage at the ring stage, even with other mismatch repair, MMEJ, and HR pathways present. These observations demonstrate distinct sensitivities to DNA damage among our different DNA repair disruptions.

### PfRad51 and PfTLS polymerases differentially impact SNP and indel accumulation post irradiation

**Figure 3.**
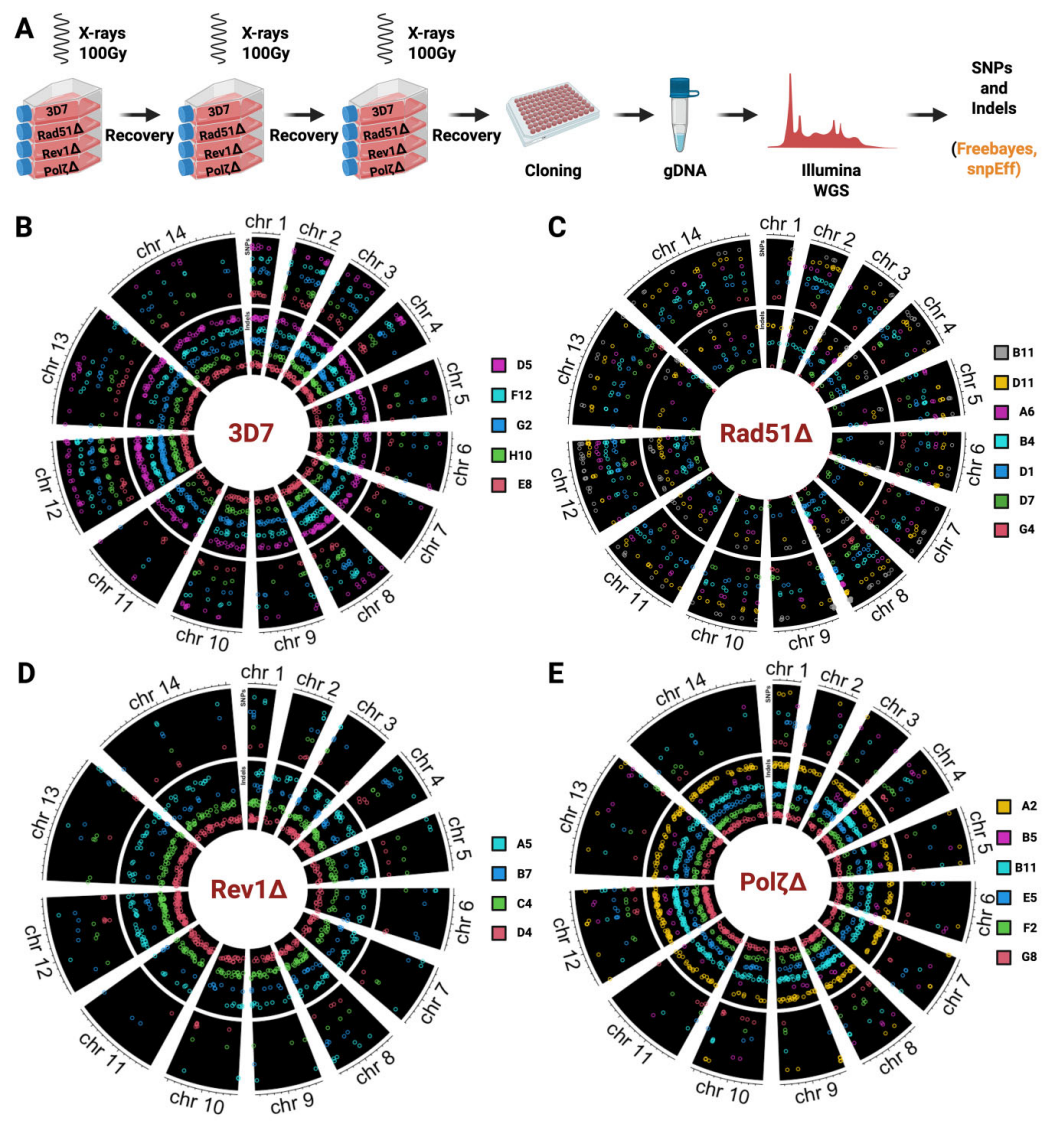
Irradiation-induced SNPs and indels. (**A**) Overview of irradiation experiments and subsequent analysis of SNPs and Indels using Illumina whole genome sequencing. Circos plots of SNPs (outer arcs) and Indels (inner arcs) distributed throughout the genome of *P. falciparum* cell lines (**B**) WT-3D7, (**C**) *PfRad51*Δ, (**D**) *PfRev1*Δ, and (**E**) *PfPol*ζΔ after 3× irradiation with 100 Gy. The different colors represent distinct clones isolated post irradiation, and the arcs represent the chromosomes.

To further characterize the functions of *P. falciparum* TLS polymerases and Rad51, we examined the genome sequence of parasites that had recovered from irradiation (Fig. 3A). Briefly, we irradiated WT 3D7, *PfRad51*Δ, *PfRev1*Δ, and *PfPol*ζΔ late-stage parasites with 100 grays of X-rays three times, allowing for parasite recovery between irradiation treatments. Clonal lines were established from recovered parasites, and genomic DNA was isolated, purified, and sequenced on an Illumina platform. The sequencing data were processed to identify single-nucleotide polymorphisms (SNPs), insertions, and deletions (indels) using Freebayes [39] and SnpEff [44], as outlined in Fig. 3A.

**Figure 4.**
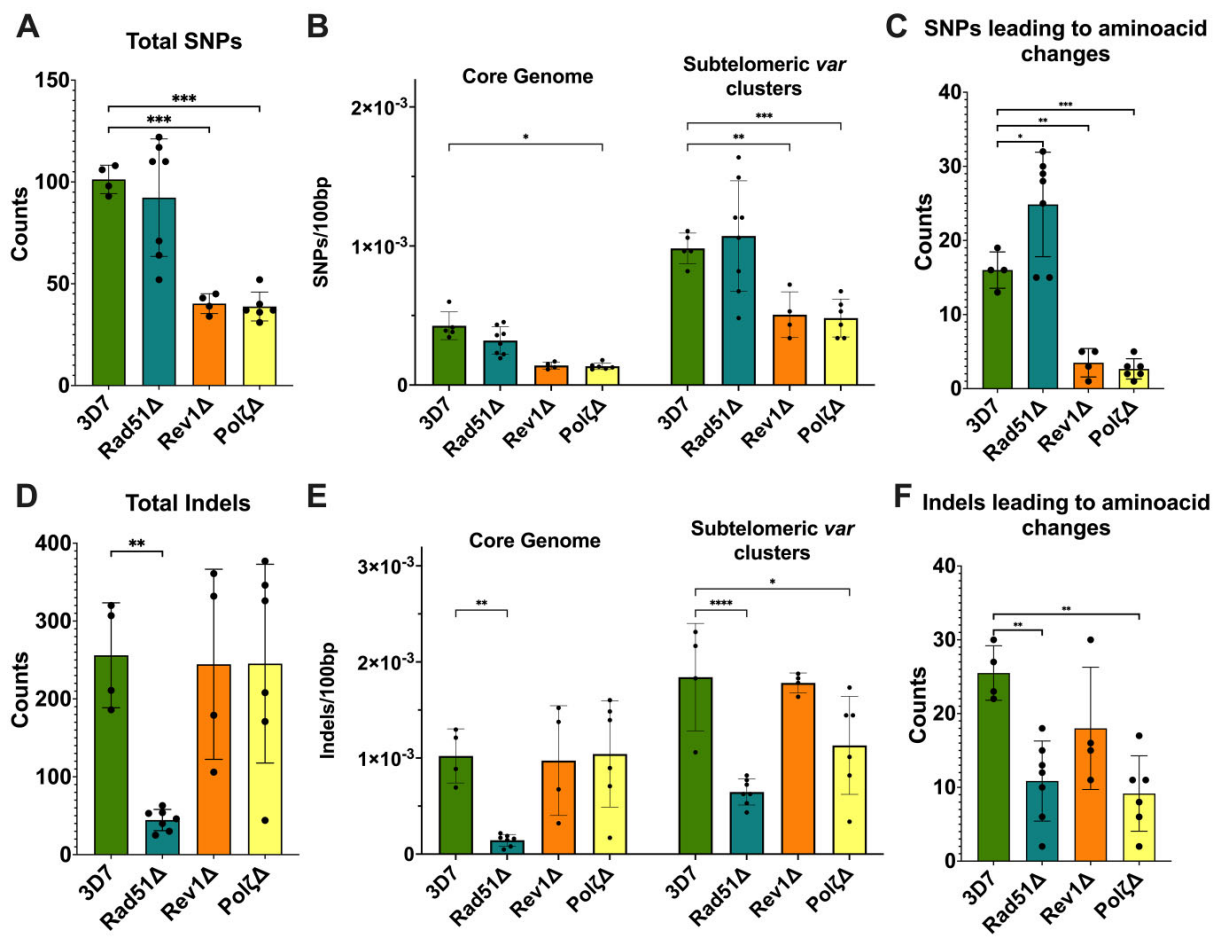
Analysis of total SNPs and indels: Quantification of total SNPs (**A**) and non-synonymous SNPs (**C**); quantification of total indels (**D**) and non-synonymous indels (**F**) from Fig. 3. Statistical significance was determined using ordinary one-way ANOVA followed by Šídák’s multiple comparisons test. *n* ≥ 4; error bars denote SD; *(*P* = .05–.01), **(*P* = .01–.001), ***(*P* = .001–.0001). Distribution of SNPs (**B**) and indels (**E**) across the core genome and the *var* clusters. Statistical significance was determined using two-way ANOVA followed by Dunnett’s multiple comparison test. *n* ≥ 4; error bars denote SD; *(*P* = .05–.01), **(*P* = .01–.001), ***(*P* = .001–.0001).

We analyzed the complete genome sequences of multiple clones of irradiated parasites from WT 3D7 and each knockout line. Data were normalized to the respective non-irradiated parental cell line. PfTLS knockout mutants were hypomutable overall and accumulated considerably fewer SNPs in coding and non-coding regions (Figs 3D and E, 4A). The identical rate of SNP accumulation in *PfPol*ζ Δ and *PfRev1*Δ supports their canonical roles working together in TLS DNA repair. Though, overall, the total number of small indels (<10 bp) was not different from WT 3D7, both *PfRev1*Δ and *PfPol*ζ Δ displayed a trend toward decreased non-synonymous indels, which reached statistical significance in the case of *PfPol*ζΔ (Fig. 4F). This modest difference between the two PfTLS mutants could be due to the more direct role Polζ has in repair in other systems compared to the more supportive role of Rev1. For example, Polζ itself can repair DSBs through a post-replicative DNA recombination pathway [71] and is recruited as a DNA polymerase during replication and recombination, leading to increased mutations [72]. Therefore, it is possible that in *P. falciparum*, the TLS polymerases might similarly have important cross-functionality responding to the repair of excised damaged bases as well as DSB repair from stalled replication forks or direct DNA damage.

The loss of *PfRad51* did not affect the frequency and number of SNPs across the whole genome (Figs 3B and 4A). We did, however, observe a small but statistically significant increased incidence of non-synonymous SNPs (Fig. 4C and Supplementary Fig. S3), suggesting that in the absence of Rad51, repair in the coding regions is error-prone. The total number of indels was significantly reduced in irradiated *PfRad51*Δ parasite clones (Fig. 4D and F). These results indicate that PfRad51 plays an important role in the repair of damage across coding and noncoding regions with different outcomes (an increase in SNP accumulations and a decrease in indels), suggesting that in *P. falciparum*, alternative compensatory pathways respond to different types of damage in the absence of Rad51.

A similar skew toward the accumulation of mutations in coding regions was also observed in other studies in which DNA replication or repair proteins were modified or deleted [73, 74]. Coding regions, specifically transcribed regions of the genome, can be more vulnerable to DNA damage due to the topological strain imposed by transcription and a more open chromatin structure, potentially leading to increased accumulation of DSBs subject to potentially mutagenic repair [75]. In *P. falciparum*, coding regions also have a pronounced increase in GC content compared to intergenic regions, which contain notable stretches of AT repeats and could affect DNA repair.

The loss of *PfRad51* resulted in significantly reduced indels (Fig. 4D), while the TLS knockouts did not show any significant changes. Given the high number of repetitive tracks in the *P. falciparum* genome, these indels are likely the result of replication slippage or, given the absence of NHEJ, MMEJ, or imprecise HR. In light of the dramatic reduction in indel formation in the absence of Rad51, we propose that Rad51 plays an outsized role in *P. falciparum* DNA repair beyond HR.

### *Plasmodium falciparum* exhibits increased SNP accumulation in the subtelomeric regions

The *P. falciparum* core genome and the subtelomeric regions are considerably different in terms of sequence diversity. While the core genome is largely conserved across parasite isolates, the subtelomeric regions are highly diverse, suggesting greater potential for recombination and mutation in these regions [4]. Previous work analyzing parasites grown under standard culture conditions found that most small indels occurred in noncoding, repetitive regions of the genome, and a small percentage occurred in subtelomeric regions of the chromosomes. In contrast, SNPs did not differ in distribution across the parasite chromosomes in WT 3D7 parasite lines [2].

We were thus interested in whether *PfRad51* or the TLS polymerases played any role in this diversification process and grouped the SNPs and indels data we obtained from the irradiated parasites into those located in the core genome versus the subtelomeric *var* clusters (Fig. 4B and E). WT 3D7 parasites displayed a higher propensity for mutations in the subtelomeric clusters post irradiation than the core genome (Fig. 4B). We observed that all three knockout lines displayed a slight reduction (statistically significant in *PfPol*ζΔ) in the number of SNPs in the core genome when compared to WT 3D7 (Fig. 4B), while both TLS knockouts resulted in reduced SNPs in both core and subtelomeric regions. In contrast, the pattern for indels remained consistent between the core and subtelomeric regions (Fig. 4E).

### PfRad51 loss abolishes radiation-induced translocations in *P. falciparum*, while TLS polymerases play a minor role in recombination events

**Figure 5.**
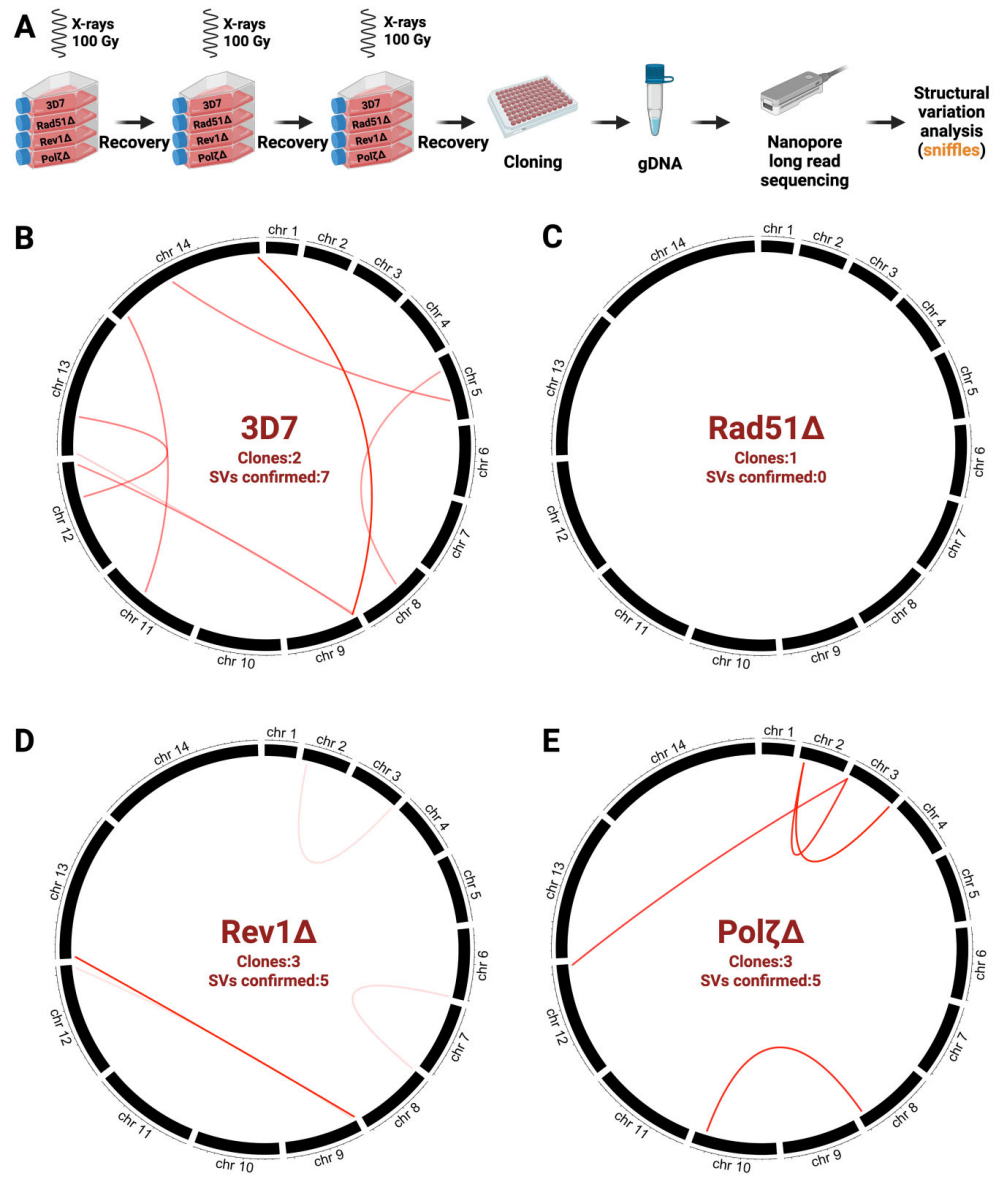
Irradiation-induced translocations/recombination. (**A**) Overview of the experimental setup for the determination of recombination events. The translocations were confirmed by visual confirmation before plotting as described in the methods. Circos plots of translocations/recombinations distributed throughout the genome of *P. falciparum* cell lines: (**B**) WT-3D7, (**C**) *PfRad51*Δ, (**D**) *PfRev1*Δ, and (**E**) *PfPol*ζΔ after 3× irradiation with 100 Gy. Each line represents a recombination event between two genomic loci identified by the breakpoints, and the transparency of the line denotes the confidence of the event based on read support for the translocation event. The confirmed SVs are compiled from all the clones tested. The visual IGV screenshots of select translocations identified in the WT 3D7 cell lines are included as examples in Supplementary Fig. S3.

We performed a detailed analysis of recombination events within the subtelomeric regions of irradiated WT 3D7 and disrupted parasite lines using genomic DNA isolated from the three times irradiated clones and Nanopore sequencing as outlined in Fig. 5A. Nanopore sequencing provides read lengths that can extend over entire recombination events, including those within clusters or tandem arrays of multicopy gene families. We expected most translocations to occur at the *var* clusters since they are more prone to recombination [4, 8]. Our analysis revealed that WT 3D7 exhibits some translocations after irradiation (∼3–4 per clone), most of which occurred between sub-telomeric regions (Fig. 5B and Supplementary Fig. S4), as expected [10]. We observed no verified translocations in a clone of irradiated *PfRad51*Δ (Fig. 5B), consistent with the anticipated complete loss of recombination in the absence of HR. However, this *PfRad51*Δ clone displayed a large deletion in the AT-rich region of the EMP1-trafficking protein gene (Pf3D7_1002100, Supplementary Fig. S5). This large deletion in a repetitive region of the core genome indicates that slip-strand repair pathways present in the parasite are Rad51-independent. The loss of TLS polymerases showed a phenotype similar to that of WT 3D7, with 3–4 translocations per clone. As most recombination events in all transgenic parasite lines occurred between two chromosome ends, these events would facilitate the generation and enrichment of novel *var* gene combinations, as previously described [3, 7–9, 76]. Recovery of recombination events may be skewed toward subtelomeric regions, as these regions are composed primarily of nonessential gene families, important for survival in the human host but not essential for growth *in vitro*, whereas mutations in the core genome are more likely to impose a fitness cost or lethality in routine culture.

### A PfRad51-independent homologous recombination pathway operates in *P. falciparum* subtelomeric regions

**Figure 6.**
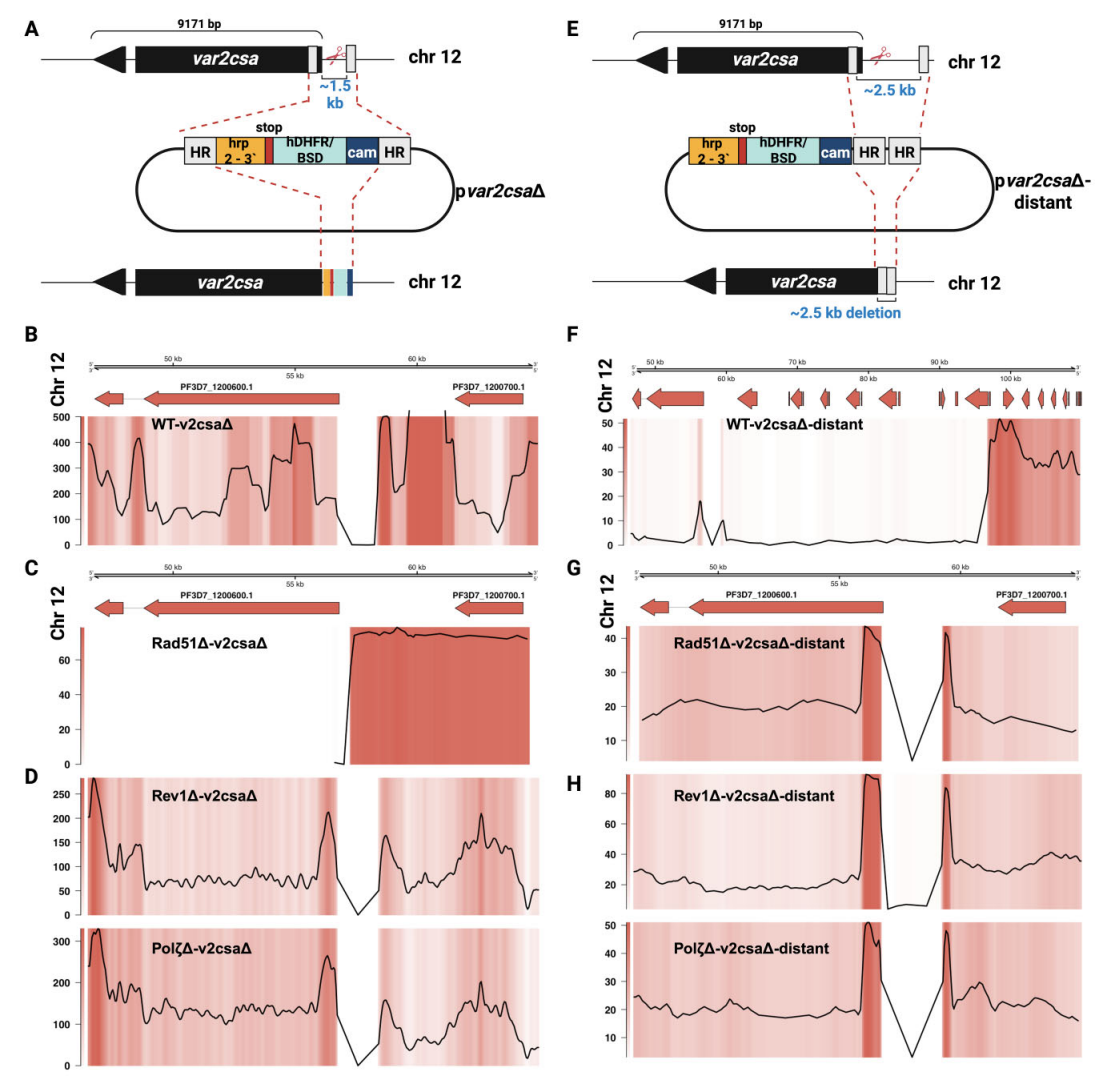
CRISPR/Cas9-mediated cuts to study homologous recombination in subtelomeric regions of *P. falciparum*. (**A** and **E**) Overview of p*var2csa*Δ and p*var2csa*Δ-distant plasmid constructs. (**B**–**D**) and (**F**–**H**) Representative coverage plots of Nanopore/Illumina sequencing results of p*var2csa*Δ and p*var2csa*Δ-distant in WT-3D7, *PfRad51*Δ, *PfRev1*Δ, and *PfPo*lζΔ. Note that *var2csa* is abbreviated as v2csa in the figures. The top panel denotes the chromosome, genomic region, and gene. The bottom panel has both heatmaps and line plots to show the integration site where the hDHFR (in the WT) or BSD cassette (in knockout lines) has been integrated or the ∼2.5 kb DNA piece [as shown in panels (G and H)] that has been lost due to HR, represented as a drop in coverage (large dip in the line plot coinciding with the white region in the coverage plot). Please note that in panel (F), the WT-p*var2csa*Δ-distant sequencing data shows a larger genomic region compared to the other samples to efficiently cover the large resection of ∼35 kb leading to TH. Shown are the Nanopore data for one transfection. Sequences from replicate transfections are included in our uploaded sequence data.

Based on previous reports demonstrating a cascade of recombination at subtelomeric regions, we compared repair dynamics at the core genome and subtelomeric regions [8, 10]. Our results above were generated from X-ray irradiated parasites, in which damage was global. Therefore, to study the effects of the loss of *PfRad51* and PfTLS polymerases on DSB repair dynamics at specific loci in the genome, we used two previously described plasmids targeting the *var2csa* locus near the end of chromosome 12 [8]. The first, p*var2csa*Δ, contained homology blocks separated by ∼1.5 kb of genomic DNA spanning the induced DSB (Fig. 6A). The second plasmid, p*var2csa*Δ-distant, contained homology blocks as a repair template, but did not include a selection cassette between the homology blocks that were ∼2.5 kb apart in the genome, resulting in markerless integration as described previously (Fig. 6E) [77]. Each repair plasmid also supplied the single guide specific for the *var2csa* gene. We chose to target *var2csa*, as it is in a cluster of multicopy genes, yet it is distinct enough from other *var* genes to reduce the likelihood of ectopic recombination events with other *var* genes, allowing for a directed DSB and alternative recombination events [78, 79]. Recombinant parasites were selected using the DHODH resistance marker on the Cas9 plasmid [28]. Each plasmid was transfected individually with a Cas9-expressing plasmid into the WT 3D7 and the knockout cell lines. It has been established in *Plasmodium* that selection for one plasmid allows for the maintenance of multiple plasmids when creating transgenic parasite lines [80].

In subtelomeric regions, DSBs can be repaired either by *PfRad51*-mediated HR or by telomere healing (TH) [8, 10, 70], a process in which the fragment between the site of the break and the chromosome end is lost, and telomere repeats are added *de novo* to the chromosome end. Parasites can survive TH as the subtelomeric regions are not essential [8, 10, 70]. In the WT 3D7 background, the p*var2csa*Δ construct resulted in efficient HR in which the two homology blocks were utilized to repair the DSB (Fig. 6B). In contrast, the loss of *PfRad51* abolished HR and forced the parasites to repair the DSB through TH (Fig. 6B). The two TLS polymerase knockouts phenocopied the WT, indicating that the *PfRad51-mediated* HR is functional in these knockouts (Fig. 6B).

Interestingly, repair using the p*var2csa*Δ-distant plasmid as a template differed from what was observed with the p*var2csa*Δ plasmid. In the WT 3D7 background, when the p*var2csa*Δ-distant construct was provided as the template for repair, we observed that the repair machinery failed to recognize the homology blocks, resulting in a large (∼35 kb) resection and TH (Fig. 6F). This indicates that despite a functional HR pathway within the subtelomeric regions, TH outcompetes HR when there is a significant distance between homology blocks. Surprisingly, we observed homology-mediated repair of a subtelomeric DSB in the *PfRad51*Δ line despite the loss of the canonical HR pathway (Fig. 6G). This was unexpected and also observed in the two TLS knockouts (Fig. 6H), indicating that when the distance between the two homology blocks was greater than 2 kb, in the absence of *PfRad51* or the two TLS polymerases, we saw the activation of an alternate, Rad51-independent pathway that can perform homology-directed repair specifically within the subtelomeric region. When we examined the individual reads that spanned the DSB site in all three knockouts, we observed recombination events that identified the first homology block but initially failed to recognize the second homology block and instead replicated the entire plasmid backbone multiple times before using the second homology block to complete recombination (Supplementary Fig. S6). To confirm this, we generated genome assemblies from Nanopore sequencing data to accurately visualize breakpoint sequences. Our reads were of suitable quality to ensure an accurate assembly in *PfRad51Δ-var2csaΔ*-distant (Supplementary Fig. S7A) and *PfPolζΔ-var2csaΔ*-distant reads (Supplementary Fig. S7B). The assembly confirmed that repair was due to HR. Detailed analysis of breakpoints was most consistent with HR, and there was no evidence of repair by other mechanisms, such as MMEJ/Alt-EJ. Sequence assemblies obtained from multiple repeated transfection experiments (three transfections each) confirmed the incorporation of multiple copies of the plasmid at the site of repair in all three of the knockout lines when the DSB targeted a subtelomeric location. Previous work described similar single crossover integration events in CRISPR and non-CRISPR-mediated genetic modifications in *Plasmodium*, often with integration of the entire plasmid as a concatemer [81, 82]. It is, however, clear that with the same guide and donor plasmid, we observed very different repair events in our WT 3D7 versus knockout parasite lines. Though the two plasmids are not directly comparable given differences in plasmid design, recombination with p*var2csa*Δ indicates that HR was possible in WT 3D7 and TLS Δ parasites, but that different constraints are placed on recombination when using the p*var2csaΔ*-distant construct, resulting in the different recombination products.

### PfRad51 is essential for DSB repair in *P. falciparum*’s core genome, while TLS polymerases are dispensable

**Figure 7.**
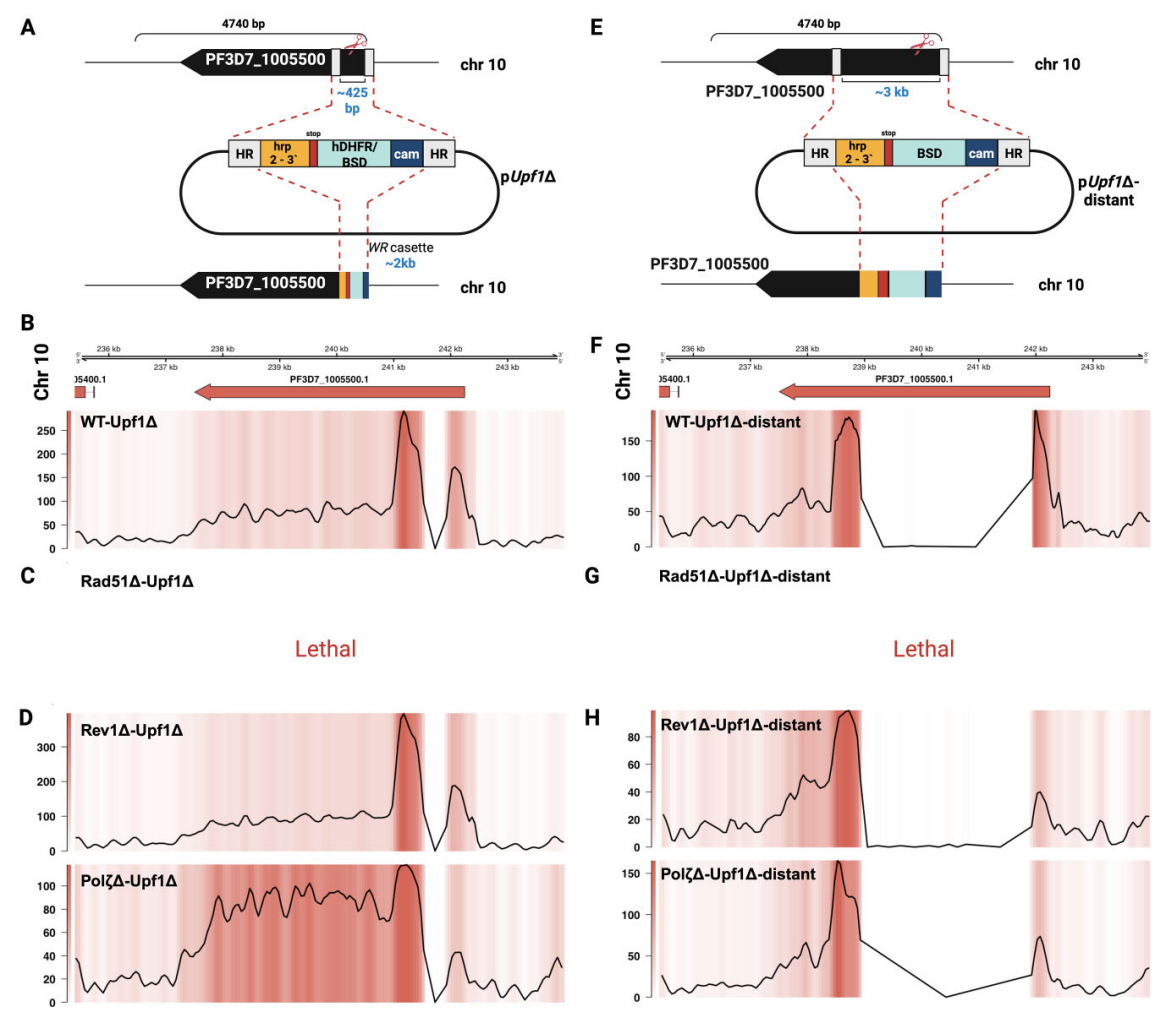
CRISPR/Cas9-mediated cuts to study homologous recombination in the core genome of *P. falciparum*. (**A** and **E**) Overview of p*Upf1*Δ and p*Upf1*Δ-distant plasmid constructs. (**B**–**D** and **F**–**H**) Representative coverage plots of Nanopore/Illumina sequencing results of *PfUpf1*Δ and *PfUpf1*Δ-distant in WT-3D7, *PfRad51*Δ, *PfRev1*Δ, and *PfPol*ζΔ. The top panel denotes the chromosome, genomic region, and the gene. The bottom panels have both heatmaps and line plots to show the integration site where the hDHFR (in WT-*Upf1*Δ line) or BSD resistance cassette (in knockout lines) has been integrated, which can be seen as a drastic drop in the coverage (large dip in the line plot coinciding with the white region in the coverage plot).

In contrast to the recombination events described earlier, TH is not an option in the core genome; thus, we chose to use a similar approach to study repair events in the parasite core genome and targeted a housekeeping gene known to be non-essential [83, 84]. The donor plasmids contained repair templates for *PfUPF1* (PF3D7_1005500) with homology blocks at different lengths from the induced DSB p*Upf1*Δ with a template ∼425 bp and p*Upf1*Δ-distant with a template ∼3 kb from the DSB (Fig. 7A) [83]. We chose to use selection cassettes in both plasmids for these experiments to increase the number of parasites that had sustained a DSB and undergone repair. The donor plasmids also supplied the single guide targeting the *PfUpf1* gene, as depicted in Fig. 7A and D. Each plasmid was transfected with a Cas9-expressing plasmid into WT 3D7 and the knockout cell lines: *PfRad51*Δ, *PfRev1*Δ, and *PfPol*ζΔ. As per standard protocols, transfections were selected for 4 days with DSM1 for the Cas9-carrying plasmid. This was done to enable isolation of a potentially diverse population of transfectants. Each transfection was repeated three times.

In WT parasites and both TLS knockout lines, we observed successful and efficient DSB repair by HR (Fig. 7B and C). Specifically, in the WT, *PfRev1*Δ, and *PfPol*ζΔ strains, we recovered parasites with the predicted double crossover event, resulting in the replacement of the sequence targeted by both the p*Upf1*Δ and p*Upf1*Δ-distant constructs (Fig. 7B, D, F, and H). This was expected, as we targeted a dispensable housekeeping gene, and as *PfRad51* is intact in all these lines, the HR pathway should be functional. As TH is not an option in the core genome, HR proceeded as expected even when the distance between homology blocks was increased (Fig. 7E).

However, in the absence of *PfRad51*, the Cas9-induced DSB proved lethal, with both donor plasmids, p*Upf1*Δ and p*Upf1*Δ-distant, unable to serve as templates for repair in the *PfRad51*Δ cell line. Thus, *PfRad51* is essential for the repair of DSBs in the core genome, and TLS polymerases have a limited or no role in standard HR in *P. falciparum*.

## Discussion

We set out to identify mechanisms of *P. falciparum* genome diversification with a particular interest in how DNA repair pathways vary at different chromosome locations and are beginning to understand how the parasite segregates its genome into a well-conserved core and highly divergent subtelomeric regions [4]. *var* gene diversity is critical to the parasite’s ability to evade the host immune response and is an important determinant of the virulence and persistence of *P. falciparum* infections. Most work to date has characterized and classified *var* gene diversity, providing interesting but limited insights into the molecular nature of the diversification process [9, 76, 85–87], whereas more recent work has begun to address the mechanisms underlying *var* gene diversification [7, 8, 17]. Analysis of recombination events showed that, in general, *var* gene recombination maintains open reading frames and preserves domain architecture, resulting in functional chimeric *var* genes, consistent with gene conversion and NAHR as the primary mechanisms for generating novel *var* gene sequences during mitotic replication [7]. This idea is further supported by work describing recombination identified in parasites subjected to irradiation and targeted DSBs via CRISPR/Cas9 [8, 10]. Both modalities of DNA damage yielded subtelomeric gene recombination consistent with a cascade of ectopic HR events leading to the formation of chimeric *var* genes [8, 10]. These ectopic HR events were primarily observed in subtelomeric *var* clusters where the repair choice between TH and HR drives diversification [10].

One of the most striking observations of our study is that *PfRad51, PfRev1*, and *PfPol*ζ are not essential for the survival of parasites and that there was no growth defect under standard culture conditions, even given the replication stress the parasite is under during erythrocytic development (Supplementary Fig. S2A). Since *P. falciparum* lacks the error-prone NHEJ pathway, we predicted loss of *PfRad51* would render most DNA breaks unrepaired and thus lethal. This contrasts with the essentiality of Rad51 in vertebrates [47] but aligns more closely with observations in unicellular eukaryotes such as yeast [48, 50]. The notable stage-specific differential sensitivity to irradiation between the ring and late stages observed in the TLS knockout parasite lines underscores the importance of stage-specific DNA repair mechanisms in *P. falciparum*, similar to the dominance of different DNA repair pathways across the cell cycle in higher eukaryotes [88]. In late stages (Fig. 2A, ∼24–36 hpi), which could be compared to the S-phase of typical eukaryotic cells [89], HR appears to dominate repair due to the availability of a second copy of the genome. In this setting, the TLS polymerases are dispensable. In contrast, ring-stage parasites are haploid and are not undergoing DNA replication and, therefore, can be considered analogous to the G1 phase in higher eukaryotes [68, 89]. In this scenario, repair of DNA damage depends on non-HR pathways and, owing to the absence of c-NHEJ in *P. falciparum*, the loss of TLS is lethal. The differences in stage-specific requirements of TLS polymerases highlight the complex and dynamic nature of DNA repair in the parasite.

Our analysis of irradiated parasite lines revealed different mutational signatures between wild type and, *PfRev1* and *PfPol*ζ disrupted lines. Although the enzymatic activities of *PfRev1* and *PfPol*ζ have yet to be biochemically assessed, the hypomutable phenotype observed in our TLS disruption irradiation experiments is consistent with their classical roles. Additional work is needed to clarify the specific functions of these polymerases in *Plasmodium*, particularly given the pronounced phenotype of parasites lacking functional TLS polymerases at the early ring stage. In contrast, the SNP accumulation in our *PfRad51*Δ line was modestly increased, particularly in coding regions. This SNP accumulation in coding regions of other DNA repair mutants, but the basis of this distribution is not well understood. Together, our mutagenesis experiments indicate that translocations and indels are majorly *PfRad51* dependent, while both TLS and HR appear to play a role in novel SNP generation.

**Figure 8.**
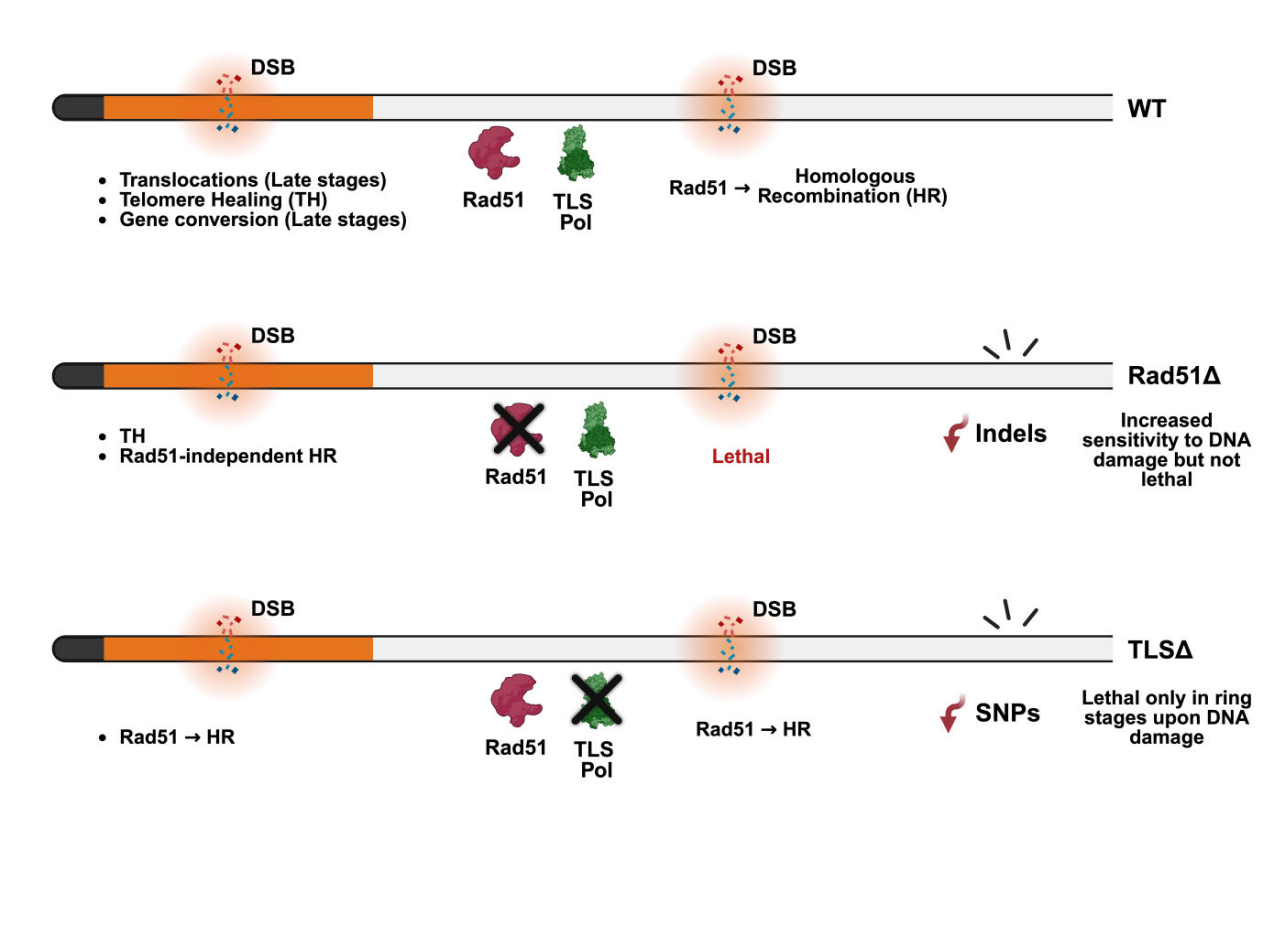
Model of a balanced genome diversification in *P. falciparum*. Summary figure with major results from this study. Our irradiation studies (global DNA damage) show that indels and translocations are *PfRad51*-dependent, whereas novel SNPs are dependent on the TLS pathway. The directed cuts using CRISPR/Cas9 indicate that DSB repair in the core genome is *PfRad51* dependent, while DSB repair in subtelomeric regions can be independent of *PfRad51;* what compensates for *PfRad51* here is still unknown. With this model, *var* gene diversification can occur through two pathways: a Rad51-dependent pathway leading to translocations and a Rad51-independent HR pathway.

While *PfRad51* is essential for repairing DSBs in the core genome (Figs 7C and G and 8), it is not essential in subtelomeric regions (Figs 6C and G and 8) where parasites can choose TH over HR. However, in the same subtelomeric region, when the distant repair template was provided, the choice of repair was HR, and to our surprise, this HR was *PfRad51* independent (Figs 6G and 8). This difference between repair mechanisms in the core and subtelomeric regions might contribute to the highly diverse nature of the subtelomeric regions and the relatively conserved core genome, leading to segregated genome diversification (Fig. 8). Future work will aim to study the yet unknown proteins involved in Rad51-independent HR. Further exploration into proteins that play a role in this process has the potential to identify novel mechanisms that enable human malaria parasites to diversify the *var* repertoire and thereby evade the human immune response more efficiently through the creation and activation of novel, chimeric *var* genes.

While our experiments provide a detailed view of potential differences in repair choices between core and subtelomeric regions, caveats remain. In particular, we recognize that in some instances, the experiments described earlier are not directly comparable since our experiments are restricted by the choice of repair templates that can be successfully cloned and maintained in a plasmid. For example, the presence or absence of the selectable marker could possibly affect the repair choice even though the transfectants were not under selection pressure. In addition, the transcription state of the gene or the loci targeted by CRISPR might also affect the repair choice. However, we are confident in our observations that there are differences in repair choice at different chromosomal locations and in the presence or absence of key DNA repair proteins.

The *P. falciparum* genome is unique in many aspects and can serve as a model for the study of DNA repair under high oxidative and high replicative stress in a haploid organism that lacks NHEJ. The evolution and regulation of DNA repair pathways in the malaria parasite can serve as guides not just to antigenic variation and drug resistance in this parasite of global importance but also to DNA repair mechanisms in other eukaryotes.

## Supplementary Material

gkaf1275_Supplemental_File

## Data Availability

All the sequencing data have been deposited to the SRA database (https://www.ncbi.nlm.nih.gov/bioproject/PRJNA1244413). All the codes for the WGS analysis performed are available at Zenodo (https://doi.org/10.5281/zenodo.15312139).

## Notes

Present address: Department of Epidemiology and Biostatics, Memorial Sloan Kettering Cancer Center, New York,NY 10065,United States

Present address: Molecular Biology Program, Memorial Sloan Kettering Cancer Center, New York,NY 10065,United States
