# Supplementary material for: Plasmodium falciparum DNA repair dynamics reveal unique roles for TLS polymerases and PfRad51 in genome diversification
Source: Nucleic Acids Res. 2025 Nov 24;53(21):gkaf1275. doi: 10.1093/nar/gkaf1275 (PMC12641262; doi:10.1093/nar/gkaf1275)

# Supplementary Data

**Supplementary Table 1**: List of plasmids generated from pL6-eGFP as described in the methods section.

Plasmid name Description

p*Rad51Δ* Generating knockout of *PfRad51*

p*Rev1Δ* Generating knockout of *PfRev1*

p*Pol𝜁Δ* Generating knockout of *PfPol𝜁*

p*Upf1Δ* Generating knockout of *PfUpf1*

p*Upf1Δ-distant* Generating distant version knockout of *PfUpf1*

p*var2csaΔ* Generating knockout of *Pfvar2csa*

p*var2csaΔ-distant* Generating distant version knockout of *Pfvar2csa*

**Supplementary table 2**: DNA sequences used in the study

**Oligo Sequence Target**

TTATTTGGTGAATTTCGTAC single guide for generating *PfRad51*Δ

ATATTAATAGTTCAAAACGG single guide for generating *PfRev1*Δ

TTATCTTTTTAATGAAAGTG single guide for generating *PfPol*𝜁Δ

TGAATGCAATCATTACCATG single guide for generating *PfUpf1*Δ

TGGAATAAAGAAACATACTT single guide for targeting *var2csa*

AACCAAGACAAAATAAAGGCCG Forward qPCR primer for *PfPol*𝜁 (AV229)

TCGTTGCTATAATTCCCATGCAA Reverse qPCR primer for *PfPol*𝜁 (AV230)

CCAAGTCGTTGCCAAGGTTG Forward qPCR primer for *PfRad51* (MH13)

TGCAGATTCGGCTTTCTCCC Reverse qPCR primer for *PfRad51* (MH14)

TCGTGTATTATCAGTAGACGAATCA Forward qPCR primer for *PfRev1* (MH71F)

TCGTTGGAAGCTTCTGGAGT Reverse qPCR primer for *PfRev1* (MH71R)

**Supplementary table 3**: DNA sequence of homology blocks used for generation of cell lines.

***PfRad51*Δ**

Homology block 1:
CTGAAACAAGCAAATACAAAAGAAGATAAGTCGCAGAAAATTTCCAATAGTAGTACTATTGATGAAATAGAAGAAGAACAGTTATATACTGGACCTTTAAAAATAGAACAATTATTAGCAAAAGGTTTTGTAAAAAGAGATTTAGAATTACTGAAGGAGGGTGGATTACAAACTGTTGAATGTGTGGCATATGCTCCTATGCGTACGTTATTGCTATTAAAGGTATAAGTGAACAGAAGCAGAGAAATTAAAAAAGGCAGTAAAGAATTATGTAATTCAGGTTTTTGTAATGCTATAGATTATCACGACGCAAGACAAAATTTAATAAAATTTACCACAGGATCAAAACAATTAGATGCCC

Homology block 2:
CTATTACATGTCAATTACCTATTGAACAATCAGGAGGAGAAGGTAAATGTTTATGGATAGATACGGAAGGTACTTTTAGACCTGAGCGTATTGTAGCTATCGCTAAAAGGTATGGTTTACATCCAACGGATTGTTTAAATAATATAGCATATGCTAAAGCCTATAATTGTGATCATCAAACTGAATTATTAATAGATGCTAGCGCTATGATGGCAGATGCAAGATTTGCTTTATTAATTGTGGATTCAGCAACGGCTCTATATAGATCTGAATATATAGGTCGAGGTGAGCTAGCTAATAGGCAATCACATTTATGTCGA

***PfRev1*Δ**

Homology block 1:
GATTCAGTGGAGGATTCAAAAGAATTAAACAATTATATGAATAACAATAGTATTAATAAAACAACGTTAGGTAAGTTTAATATGGAAAATAAAATGGAATATAATTATACTTCTATTAACGAACATAATAATAATAATTATTATATAAATAAGATACATAATGATTCTGTTTTTAAATATCAAGATATGGATAAAACACCTATGAAAAATAACATAGATAATAATAGTAATAATAATAATAATAATAATGATAATAATAATAATAATGGTAATACATATTTGTATGATAATTATCTAGAAGACCAATTTAATCAAACAATAAAAAATCGATATAGTTGTACTCCCAATGATCAAATAAGCACTTGTACAAAAAGTTTTAATACATGTATAAAAACATATGAAGAACAAGCTTCTTCAAGTTGTAAAAA

Homology block 2:
GACCAGCAAAGTAACACACATTATTAGTAATAACATGGCATTAGGTTCTAAGAAATATATGGATTATAAAAAAGCTATAAAAAAATCTAAAGTCTTCATAGTTATAGATCAATATATTTTCGATTGTGTAAACATGCAATGTCGTTTACCTGAACAATCATATCTACCTTCCATGTTACGTTACAATTGTCATCAAATAACCGAATATTTTTCCTTAAGAAAAAAAGATAAGGAACAAAACAAAAAAATGCAAAACAAAAAAATACAAGACAAAATAAATAATGATCAAAATTTTTTGAAGGAACATAAAGAAGGTG

***PfPol****𝜁***Δ**

Homology block 1:
TGAGAAAGGATGCATTGCATCATTCGAAATTAATAAAAAGAGAAAAGATGTGGTTCGTTATTCTTTTAAGAAGGAACCACCTAGAATAAACAAATGTTATGCCGTTATTAATAATTTAGTTGATCATATAAGTGGAAGAGATAACAAAAATATGAAAGAAAAAATGAAAGGAAATATTTATAATAGAATACATGACAATATAGAAGATGAGAATAAAGAAGATACATCAAAATTTGAATATATAGGAAAAGAAAATCATATGGAAAATAAGGAAAATATAAGAAAGCAATATGAACAGATCAAATCAGATAATATGAAGAAAAAAATAAATATAAAATATGGAAATATATTTTTTCTAGAAATATTAACCGAAATAAAAGATGAAAATTGTTATTCTTCAGATTATAACCAAGACAAAATAAAGGCCGTTTTTTATATAGTGAGGGAGGAAAGACTTATG

Homology block 2:
GGAACAAATGAAGTCAAATTAAATGAATCCAAATTAAATGAATCCAAATTAAATGAATCCAAATTAAATGAAGAAGAATTAAAATTGTGTAAAGATATGTATCATATAGAAGATGTGAACCTTTGTACAAATAATTGTAGAAGTATAGAAAAAAATAGGGATAATCTAATAAATGATAAGAATGTGATTAATAAAAAGAACGACGATACATATGGAAGTAGTAAAGAATTATGTTGTAGTAAAAATGGTTATCATACAAATAAAATAATAAAAGAAATAACAAATAATGATATAAAAAAGGTGAAGAGAACGTTTTTTGATTTTAATATAAATTATAATAACGTTAATATATGTATTGTAGAAAATGAAAGAGAACTTATTCAAAAGTTGATTAATAAGATATTGTTTTATTCTCCGCTTAGTATTGTTTCTTATGAAAATGATAAATATAATATAAATTATATAAACCAAAGATGTTTAGCTTTAGATATAGG

***PfUpf1*Δ**

Homology block 1:
CATTATCCACGTCGTGATCTTCTAACAAAATCTCCTTTTCCATATCATCTTGTAAACTCTTACTTTTGTGTTCATCAAAAGGACCGACATGTTTTTCATATAAGGATATATAACTAGATAAACAAGGATCTCTACAAATAATAACAACAACACCTTCTTCGGATGTCGGAAGGAAACCTAACAAAAATACATTTCTGCATGCACAGTTATAACATTCTAAAATTGTTTCACCTAATAAACTATTTTTATGTAATCTAATTTCTTTATGTTTTGATCGAACTAAATGTGTAACAATGTGACTACCACAAGTACCATAAGAACCATTACAAAACCAACGCTTACAATTATTACATTGAACAACACTATCAATAGAATCAATTTCACAATATCTGCATCTATAATATTTTAAATCATCTTTACTTTTTTTATTTTTATATCTATAATTATTACCTTCTTCATAAAGTTCATTATGTGTGTCATCCTT

Homology block 2:
ATTCCGTATCTTCCAAACTTTCATTTAACATATCTAACCTCTTTTCCTCTATTATTTTACATGGAGAATATTCCGATTTTTTATTTTTCCCATGTTTTTTTGTAGGTTTTATATTTGTATTTTTTTTTAACACCTCTTTATTACATGTATCCTTATCGTCATTAAATTTTTGTTGTGTATCTTCTGAAAAATAGTTTTCTTCTTTTATTTTTTTTTTATTCGTATCCTTAGAAATACAAAAATCGTACAGTTCGTCAAAATTATCTACATGAATATTATTATAATCGAAAGTTTTATCCATAGTTTTTCATACGCCCCTACAGATGCTTTTATAAATAAATAAATAAATATATATATATATATATATATATATATTATATATATATATAATTATATTTACTCAATTATATAAATAACCATTACAGCTATATAAAACTAATTTCTTTTTCTCCTTTTATTTTGTATGGTCTAACATATAAGGTGTCCTATACACACTCCA

Homology block 3 (distant):
GGTTTCAAGTTAGCTAAACAACCTTCTACAATTAAATCTTTTTTTTTAAATTGACTTAATAAATTGATCCATACTGAATTTACATTAGTTATGGTTTCATTTGCATTAATTTTTTCTCTGGATATAAAATGATGTCTTGATAAAACTTTTGCATTTCCACATATAATTAATCCATATTTTGCTCTGGTTAAGGCAACATTTAATCTTCTAGGATCATTTAAAAACCCTATACCTAATTTTTTATTAGATCGAACACATGATAATAATATAAAATCTTTTTCTCTTCCTTGGAATGCATCTACTGAAGCTACTTCTATATCCGACGAATTTTGAAAAGAAATATTTTTTTGAAATAATGAAGTAATATATGCTCTTTGTCCTTCATAGGGTGTTATAACACCTATTTGTGATGGTTTCAAACCACATTGTAATAAGGTACGAACTAATTTTTCCATATTAGAAGCTTCACTTCTATTAAGATAACTTGTACCTGATGCA

***Pfvar2csa*Δ**

Homology block 1:
CCACCTTAAGAATTGAGGGACATAATCTAATTTGGTAGGAACCTTTTCTTCATAATGGCCACATTTGCGACACAATTCAAGTTTATTGTCTCCATTAGATTTTCCAGATGTTCTCCATCCACGTTTTATGAGTAAATCGTTACTTCGCGCACCACAAGTAATAACTTCCCACACCTTTTGTCTATTAGCATTCCACCAATCTTCTCGTAATTTTCTATAATTTTGGTCCTTTGGGTATTTATCTTGAAGTACCTTGTCGTTTTCTCGTATTTTTGCAAACATTTGTTTTAAATTTTGTTCTAAATTACTATTAGTACCTTTCCATAGATCTGTACCTCTAATAATATCTGCAATGTCAGCAAAACTTCTTTCTAATGCATTACAAACATTGGAACTATTTGTATCTGGATGATTCTGCACTATTCTTTCTCCTTCATTTCTGGCCGTAAGTAATACATCTGCCAAAAATGCATGTTTATCCCTAATTTTATCAACATTTAATTTTTCTAAATTGTTAATGCACATACGTTGTCTTCTCGGAGGAACAAATACATTTTCAGGTTTTTCACTTACTTTAGATAAAATTATGGCACATTTCCATTGGTCATTATCGCCATAAGGAGGTGGTATTCTATCACAAGGATCATTTGTTCCAGAATCTGAATGATTCACGGTAATTTTGCAAATATTTTTTCTTAAGTAATATCCATCACCTCCACTTCCATAGTACTGCACTTCACTAGGATCAGCTTTCAACGATTGGTC

Homology block 2:
GAAGCAAATAAAGTATTCCATTTCTATCTATACACTCTATAAAATAAATAAACATAAAAACATTTACTATAATTGAATATCTTATATATATATGGTTAAATATCAATTAAAAAATATATATATATGTATGTACTATTATGTACTATATGTATTCCATTAAATCTAAATATAATTTTATGTCGTATAATAAAAACTAGTATATGCTATTCTTATTTATAATATGTATATAGTTATAATTTTATTTTATACATAATTTTTATTCTTTTTAACTTATAAATATACCTTTATTATTTTAGAATGTTATTATAATAATTATTATAATTTAAAATATAATTTCTTCTTTTTAATATGGCATGTTTTTATTTTTATTATATTTTAAGTAGTTTTATATATTTCATATATATAATTATAATTGAATATATATATATATATAGATATTTAAATGAGTTCAATTAAAAACAAAAGTCAAAATAAAT

Homology block 2 (distant):

GAATTTCATACAAATATTTTTTTATATCTATATTTGTTATAAATATATAATACTTTAGTTATATTTATAATATGTTTGTACTTTATTTTCAGATATTTTTATTATAAATAATATTATATTATTAGTTAAAGTTATGGAATTATATATATTATAAAATTATATAAATTAAAGTTATACCCTATAATAATACTACCATAATATGTATATATATACATATATATATATATATATATATATATATATATATGTGTGTATAAGTGTTTTGTAATTATATTCCTTTTTCCTTGTTTCTTTAAGCGTTCATG

**Supplementary Table 4**: List of cell lines and clones and type of sequencing done

Cell line/Clones Type of Sequencing Ref

WT(3D7) Illumina/PacBio This study(10, 69)

*PfRad51*Δ Illumina/Nanopore This study

*PfRev1*Δ Illumina/Nanopore This study

*PfPol*𝜁Δ Illumina/Nanopore This study

WT-3x-irradiated clone D5 Illumina This study

WT-3x-irradiated clone F12 Illumina This study

WT-3x-irradiated clone G2 Illumina This study

WT-3x-irradiated clone H10 Illumina This study

WT-3x-irradiated clone E8 Illumina This study

*PfRad51*Δ-3x-irradiated clone B11 Illumina This study

*PfRad51*Δ-3x-irradiated clone D11 Illumina This study

*PfRad51*Δ-3x-irradiated clone A6 Illumina This study

*PfRad51*Δ-3x-irradiated clone B4 Illumina This study

*PfRad51*Δ-3x-irradiated clone D1 Illumina This study

*PfRad51*Δ-3x-irradiated clone D7 Illumina This study

*PfRad51*Δ-3x-irradiated clone G4 Illumina This study

*PfRev1*Δ-3x-irradiated clone A5 Illumina This study

*PfRev1*Δ-3x-irradiated clone B7 Illumina This study

*PfRev1*Δ-3x-irradiated clone C4 Illumina This study

*PfRev1*Δ-3x-irradiated clone D4 Illumina This study

*PfPol𝜁*Δ-3x-irradiated clone A2 Illumina This study

*PfPol𝜁*Δ-3x-irradiated clone B5 Illumina This study

*PfPol𝜁*Δ-3x-irradiated clone B11 Illumina This study

*PfPol𝜁*Δ-3x-irradiated clone E5 Illumina This study

*PfPol𝜁*Δ-3x-irradiated clone F2 Illumina This study

*PfPol𝜁*Δ-3x-irradiated clone G8 Illumina This study

WT-*Upf1*Δ Illumina This study

*PfRad51*Δ-*Upf1*Δ Illumina This study

*PfRev1*Δ-*Upf1*Δ Illumina This study

*PfPol*𝜁Δ-*Upf1*Δ Illumina This study

WT-*Upf1*Δ-distant Illumina This study

*PfRad51*Δ-*Upf1*Δ-distant Illumina This study

*PfRev1*Δ-*Upf1*Δ-distant Illumina This study

*PfPol*𝜁Δ-*Upf1*Δ-distant Illumina This study

WT-*v2csa*Δ PacBio (8)

*PfRad51*Δ-*v2csa*Δ Nanopore This study

*PfRev1*Δ-*v2csa*Δ Illumina This study

*PfPol*𝜁Δ-*v2csa*Δ Illumina This study

WT-*v2csaΔ*-distant Nanopore This study(8)

*PfRad51*Δ-*v2csa*Δ-distant Nanopore This study

*PfRev1*Δ-*v2csa*Δ-distant Nanopore This study

*PfPol*𝜁Δ-*v2csa*Δ-distant Nanopore This study

**Supplementary Figure 1**: *var* clusters in the 14 chromosomes of 3D7 *P. falciparum*


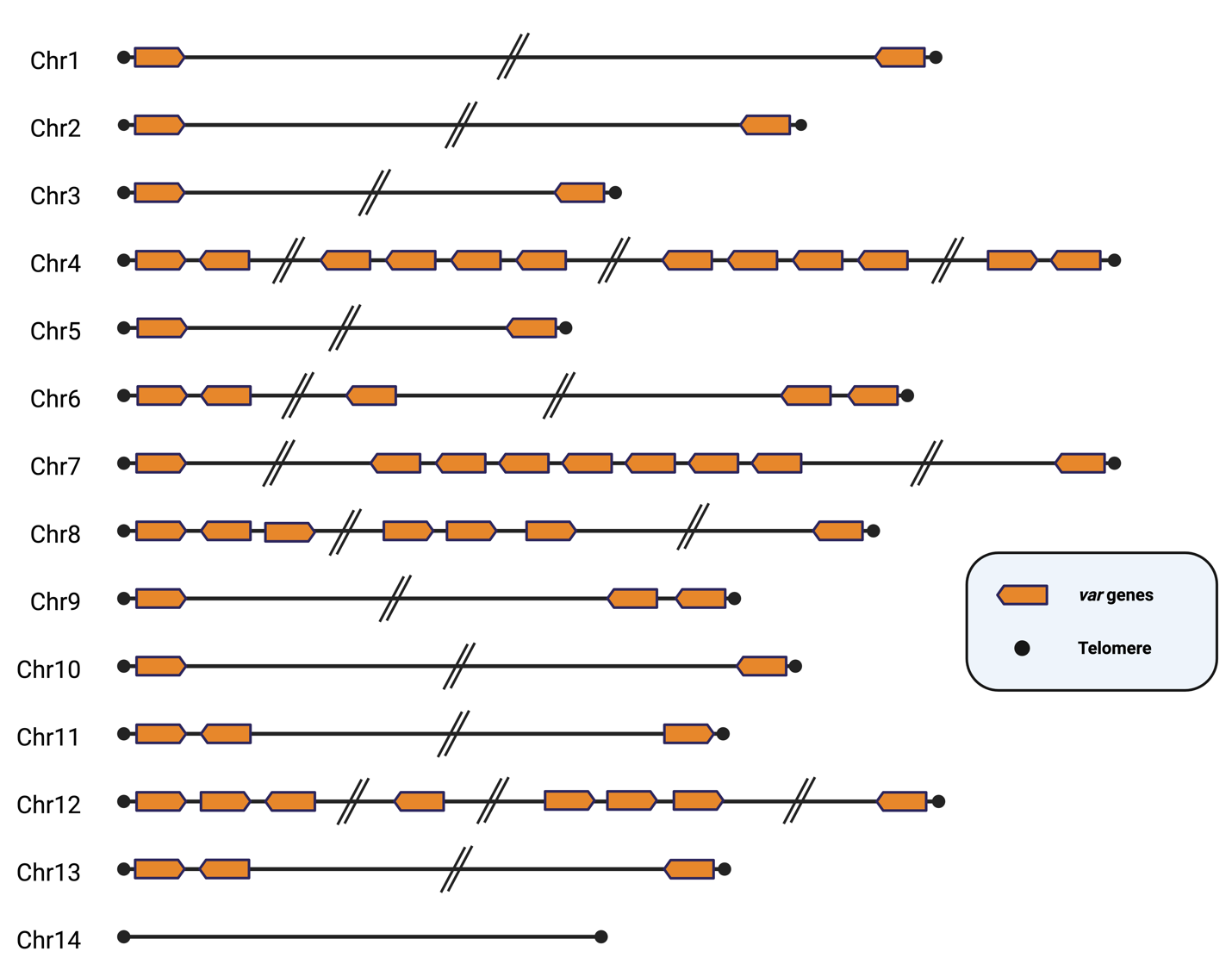


**Supplementary Figure 2**: (A) Depictions of gene disruptions with the single guide site, homology blocks (HB) and the targeted domains demarcated. Donor plasmids were designed to integrate by double crossover to disrupt our target gene and to interrupt any potential residual transcription. The purple arrows depict the primers used for RT-qPCR. The primer sequences are listed in supplementary table 2. (B) RT-qPCR data showing absence of transcripts in the knockout of the respective knocked out genes (n=3). RT-qPCR was performed as described in the materials and methods section. (C) Representative growth curves of non-irradiated knockout cell lines from this study (n=3). Highly synchronized (~98%) ring stage parasite cultures were grown from same initial parasitemia and cumulative % parasitemia was calculated for ~10 days. All the cell lines grew at a similar rate as the WT 3D7. The y-axis indicates log10 of cumulative % parasitemia. (D) Growth curve of 100 Gy irradiated ring stage (0-24 hpi) and late stage (24-48 hpi) parasites in percent parasitemia (number of infected cells/total red blood cells). The error bars represent SD (n=3). In addition to the irradiated lines, the dotted lines represent the non-irradiated parental lines allowed to grow until they reach nutrient limit and crash.

A


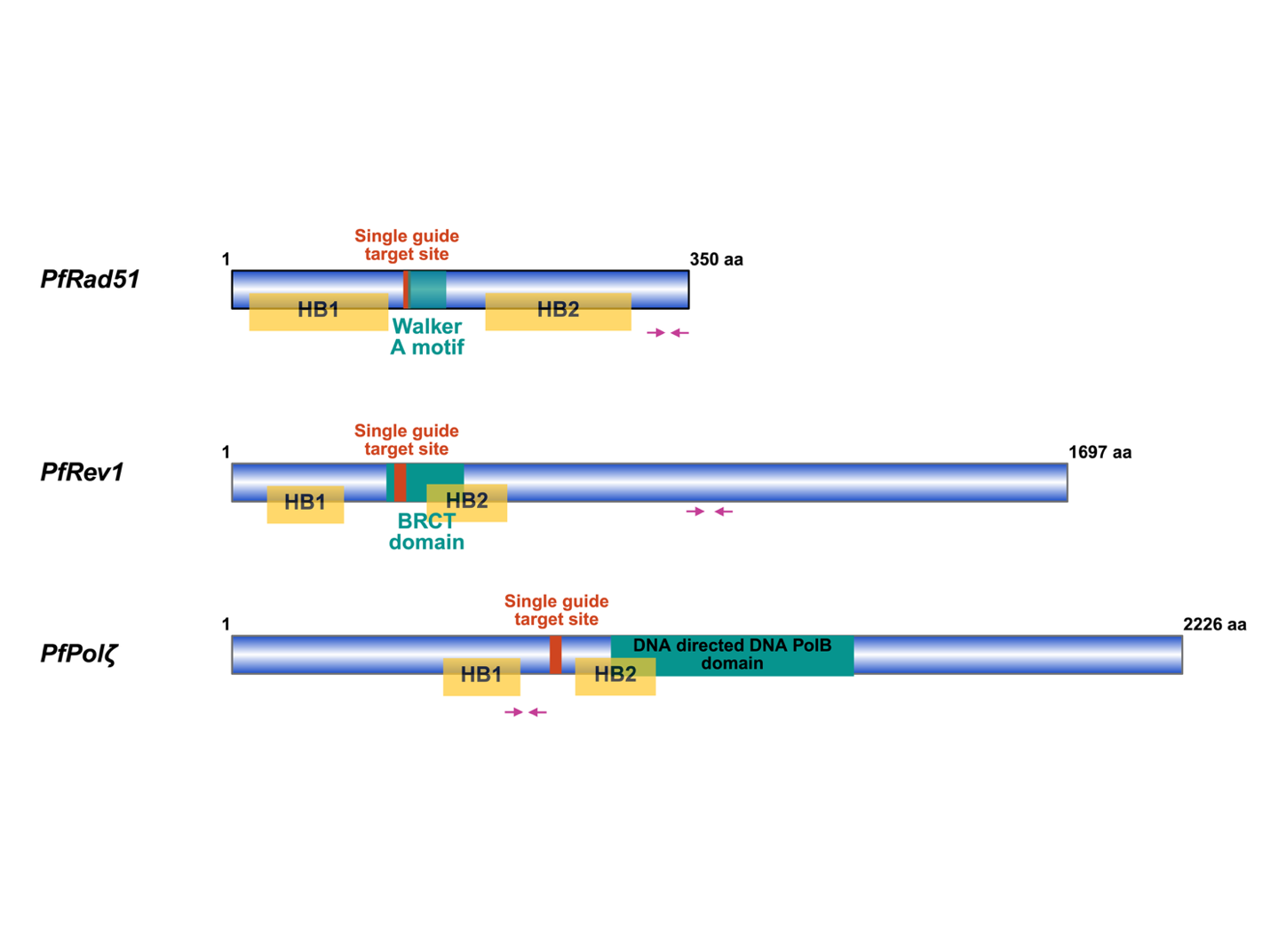


B

C

D

**Supplementary Figure 3**: Distribution of SNPs across genome: Distribution of SNPs across the genome based on the location. Upstream and downstream of the coding regions, exons, introns and intergenic regions expressed as percentage. Statistical analysis is performed using One way ANOVA followed by Šídák's multiple comparison test. *(*p*=0.01-0.05),**(*p*=0.01-0.001),***(*p*=0.001-0.0001).

**Supplementary Figures 4**: IGV screenshots of Translocations in irradiated WT 3D7 shown in Fig 5. The mismatches are highlighted with colors using the ‘show mismatch’ toggle in IGV. The pink colored reads depict the forward reads while the light blue colored reads are reverse reads. The black box indicates the SV observed in the chromosome and indicates the alignments that identified the breakpoint (BND). The boxed reads depict the translocation event, where only part of the read aligns to the chromosome (represented by pink and blue reads), and the other part of the read misaligns (represented by the blue, green, and red marks indicating misaligned bases of the read), as indicated by the supplementary alignment tag for the read.


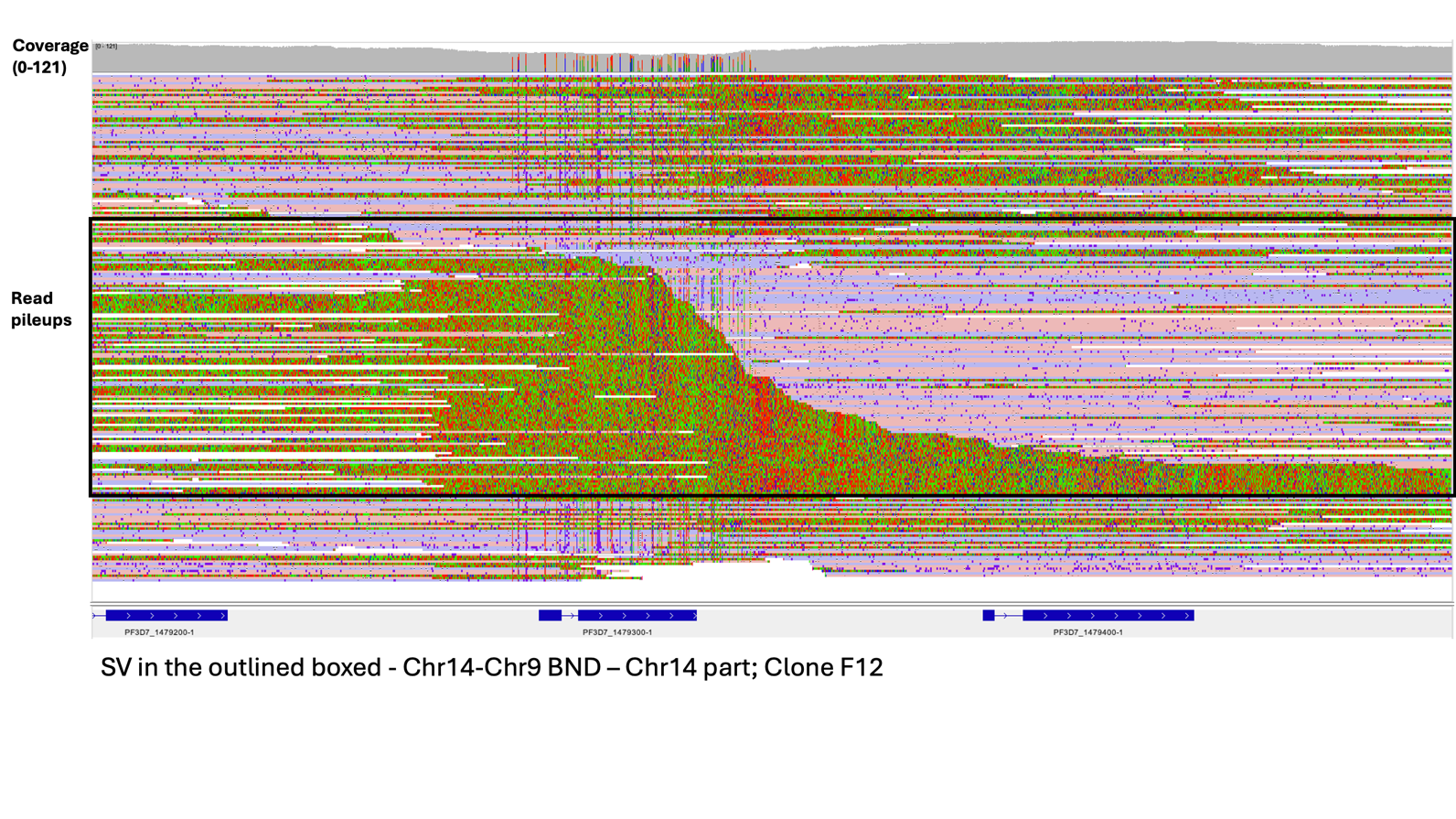


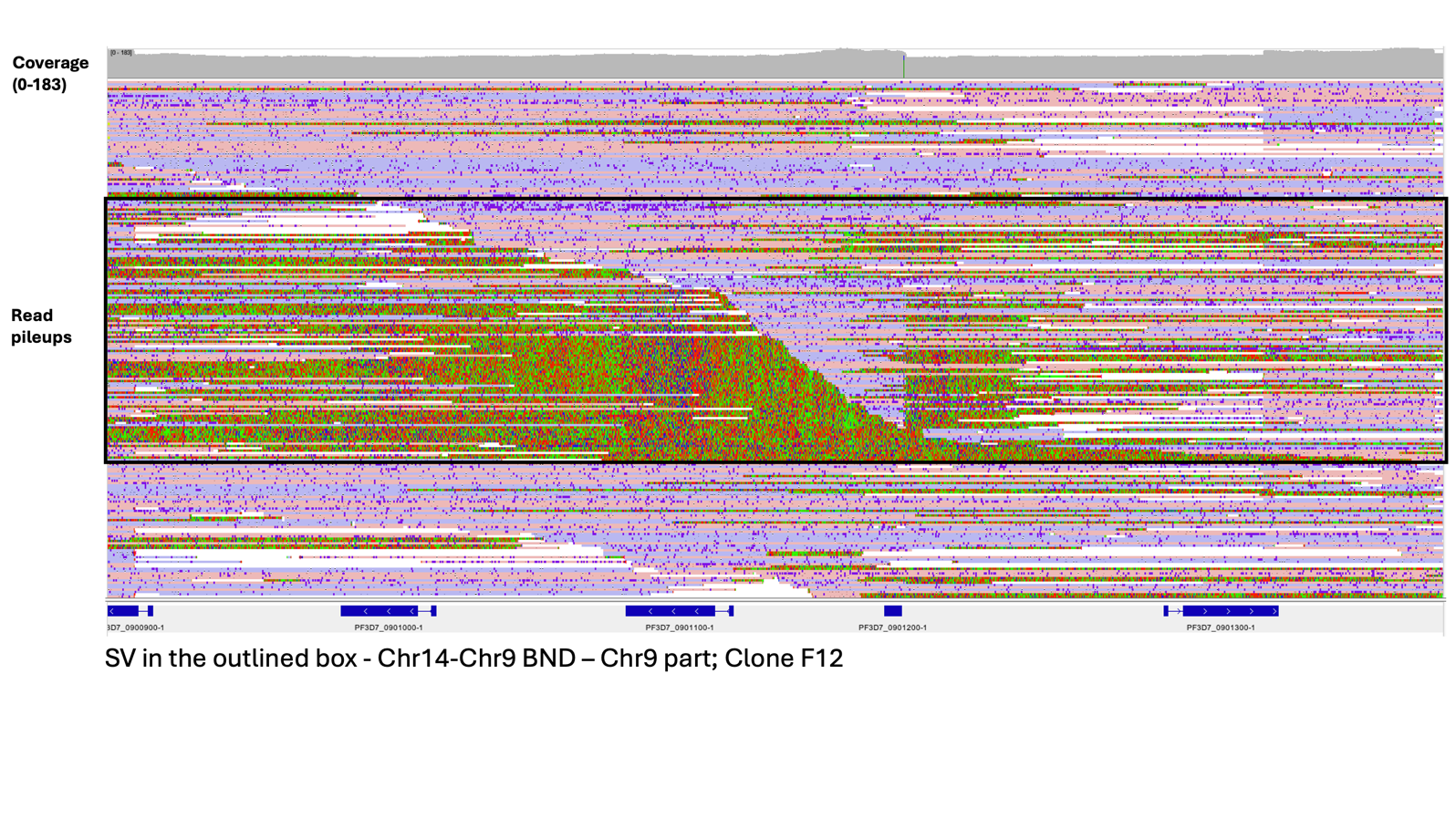

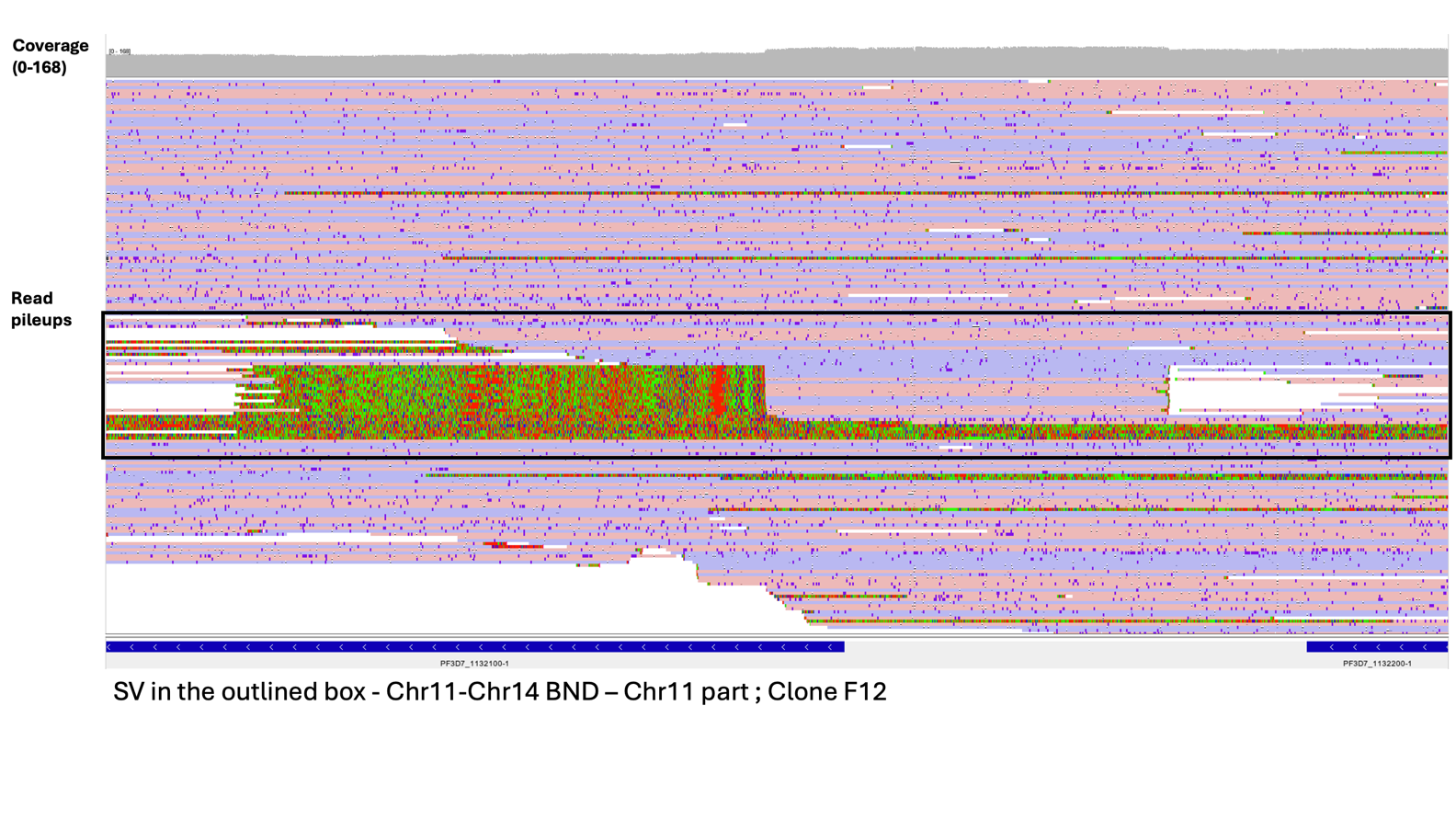

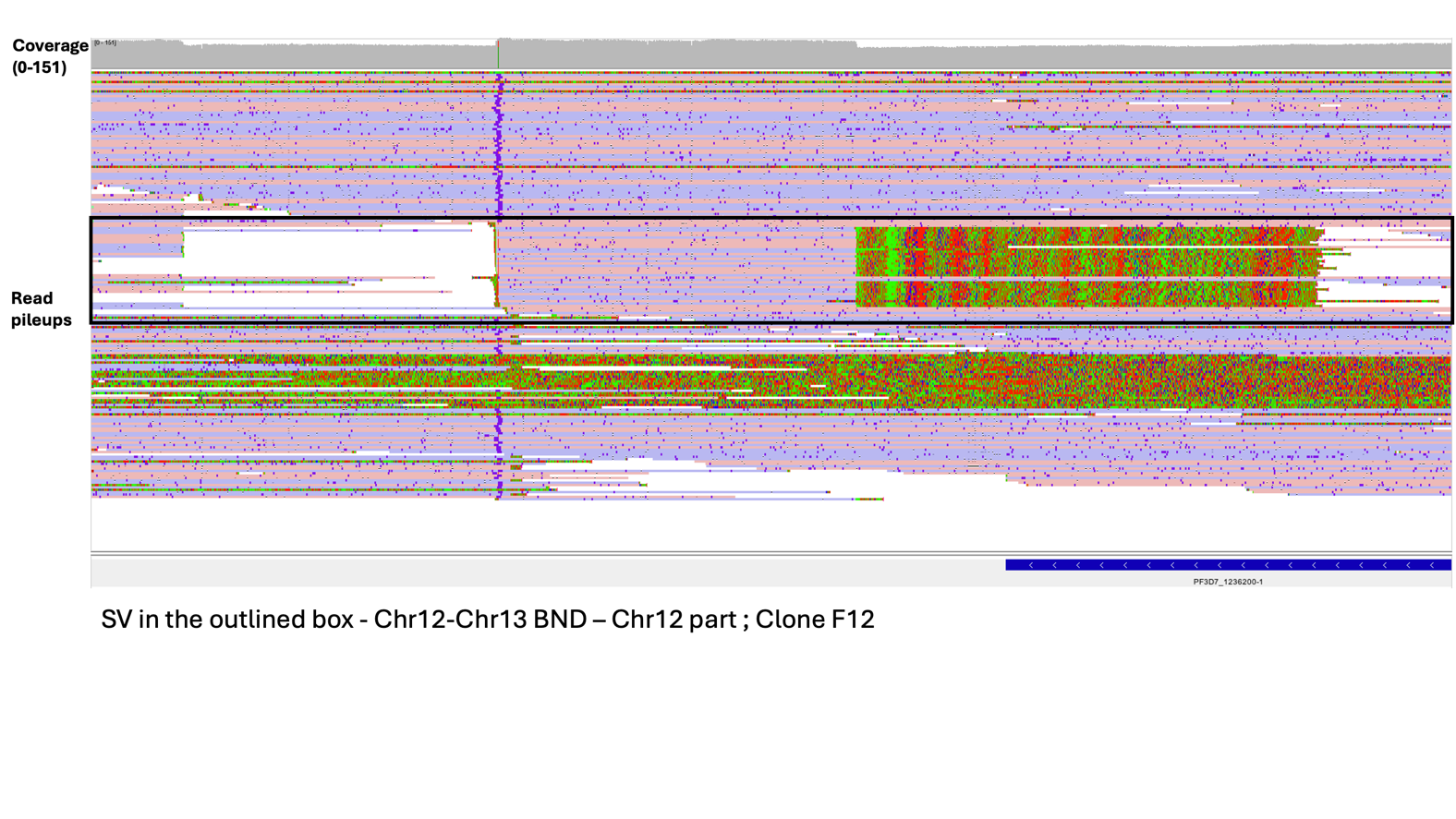

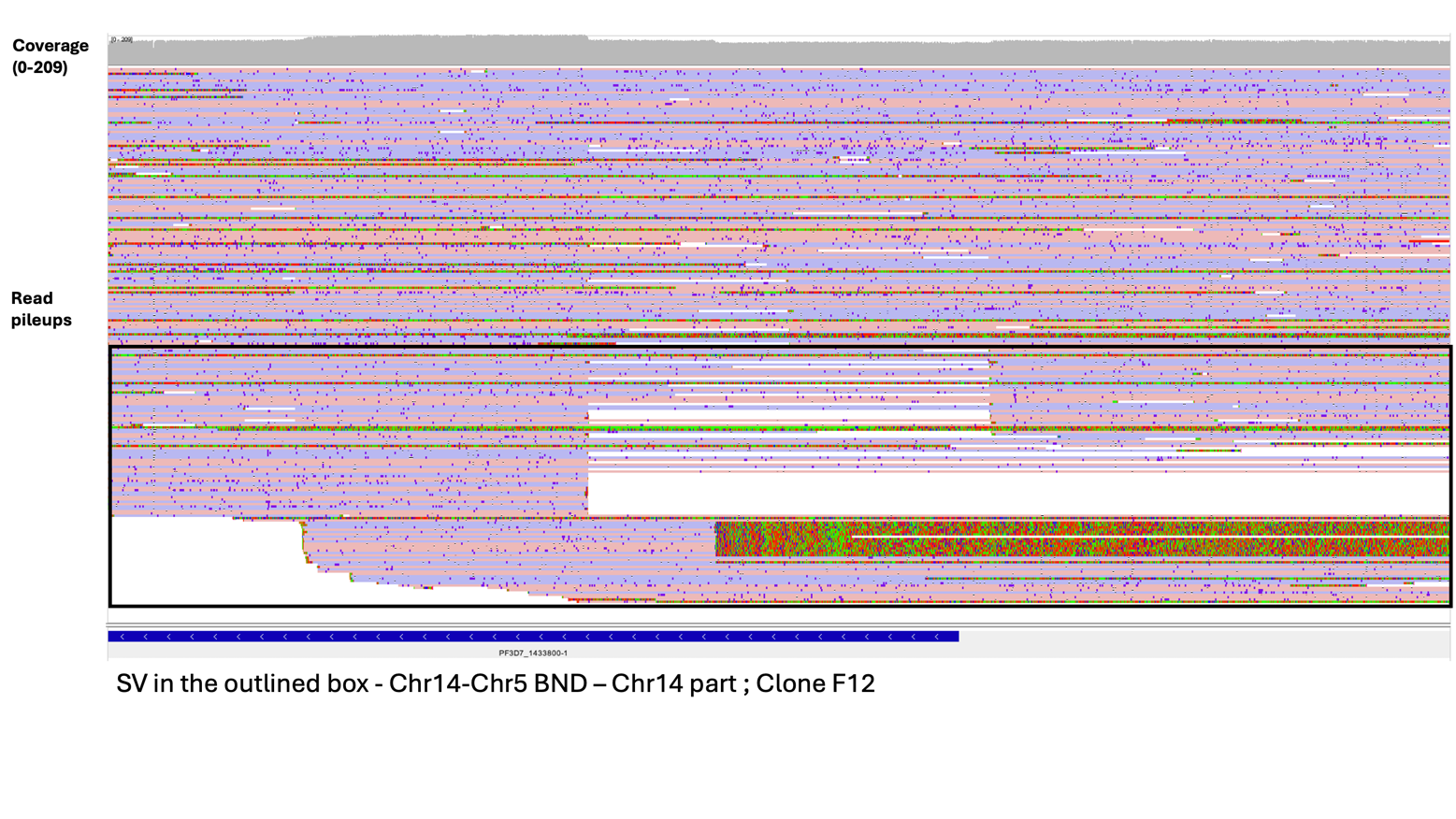

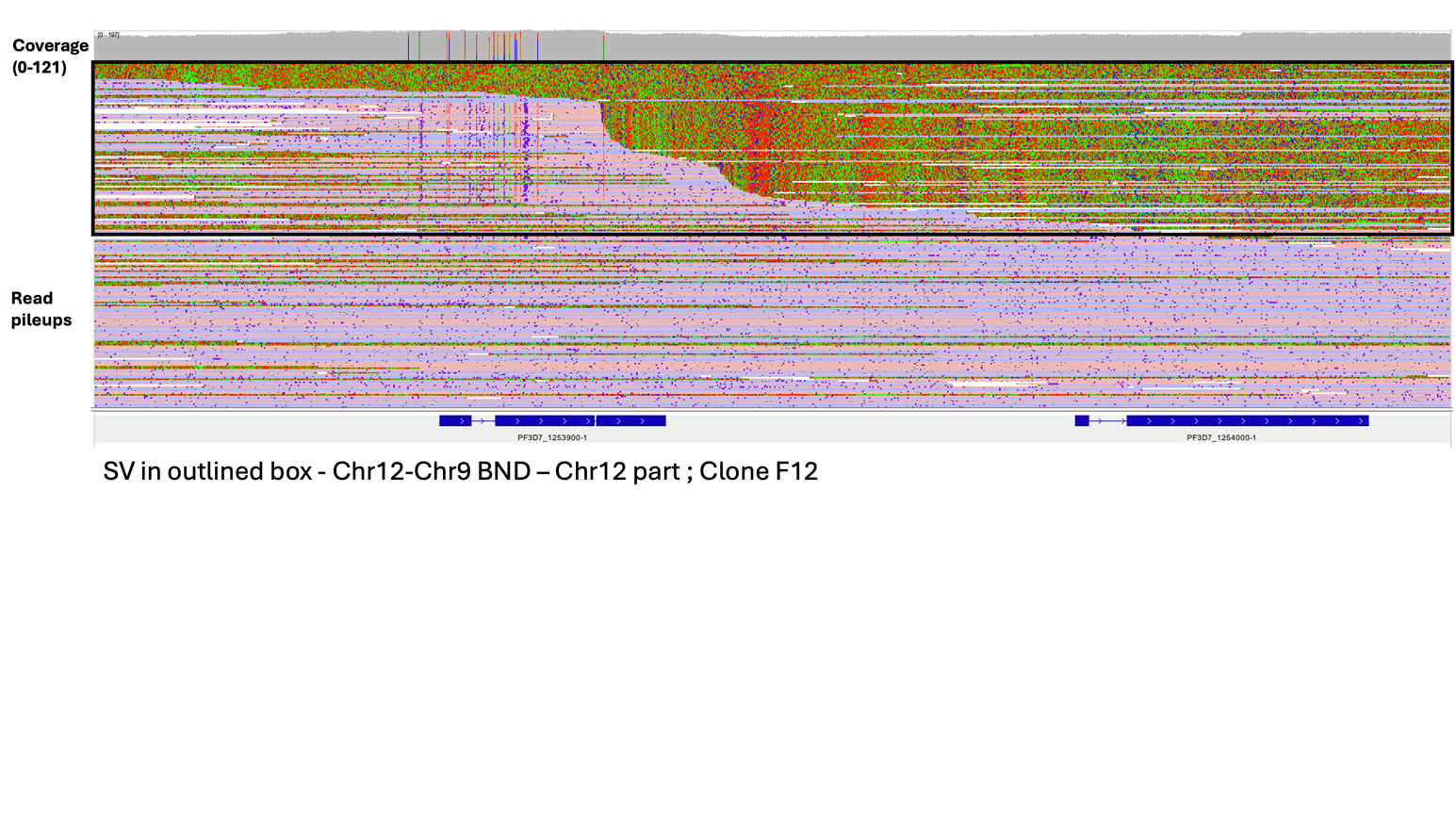

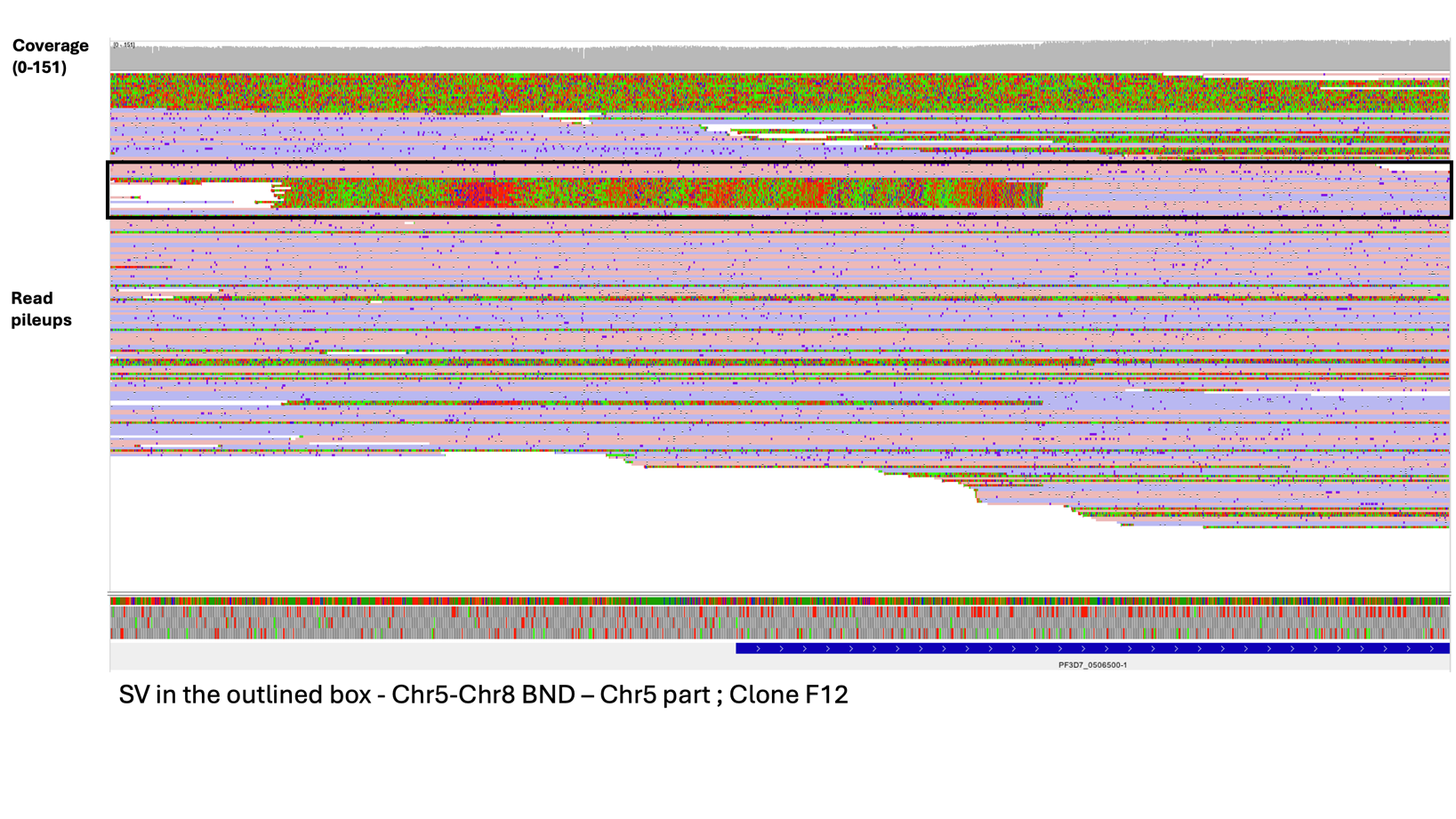


**Supplementary Figure 5**: IGV screenshot of the read pileup of *PfRad51*Δ-D9 clone at the EMP1-trafficking protein locus showing a large deletion in the repetitive sequence blocks. The pink colored reads depict the forward reads while the light blue colored reads are reverse reads. The dip in the coverage marked by the outlined box shows the deletion.


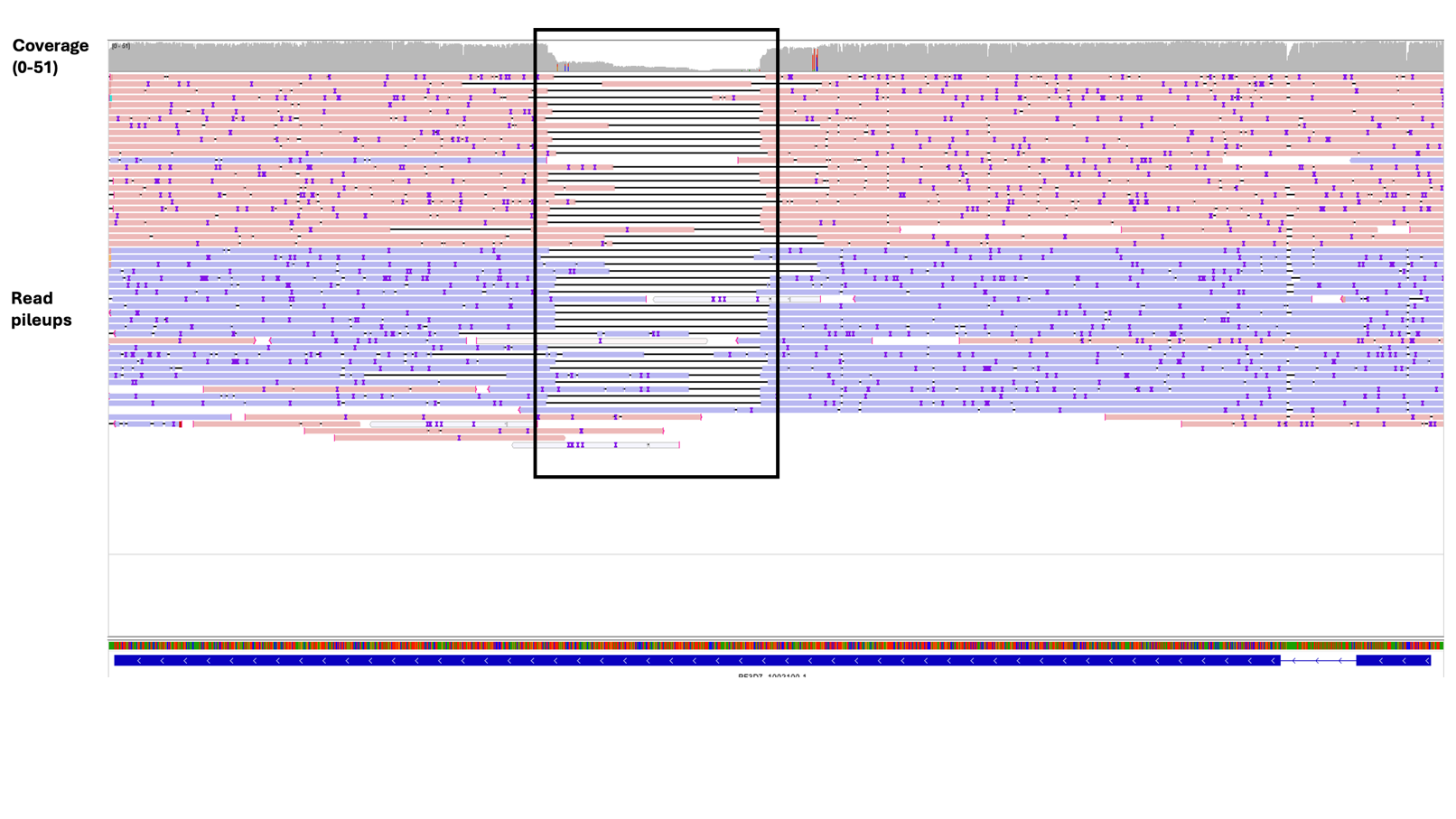


**Supplementary Figure 6**: IGV screenshots of the read pileup of distant knockout constructs. The mismatches are highlighted with colors using the ‘show mismatch’ toggle in IGV. The pink colored reads depict the forward reads while the light blue colored reads are reverse reads. In each figure, the gap between the two homology blocks (HB1 and HB2 represented by the two peaks in the coverage plots) show the loss of the genomic region which is replaced by the plasmid backbone.


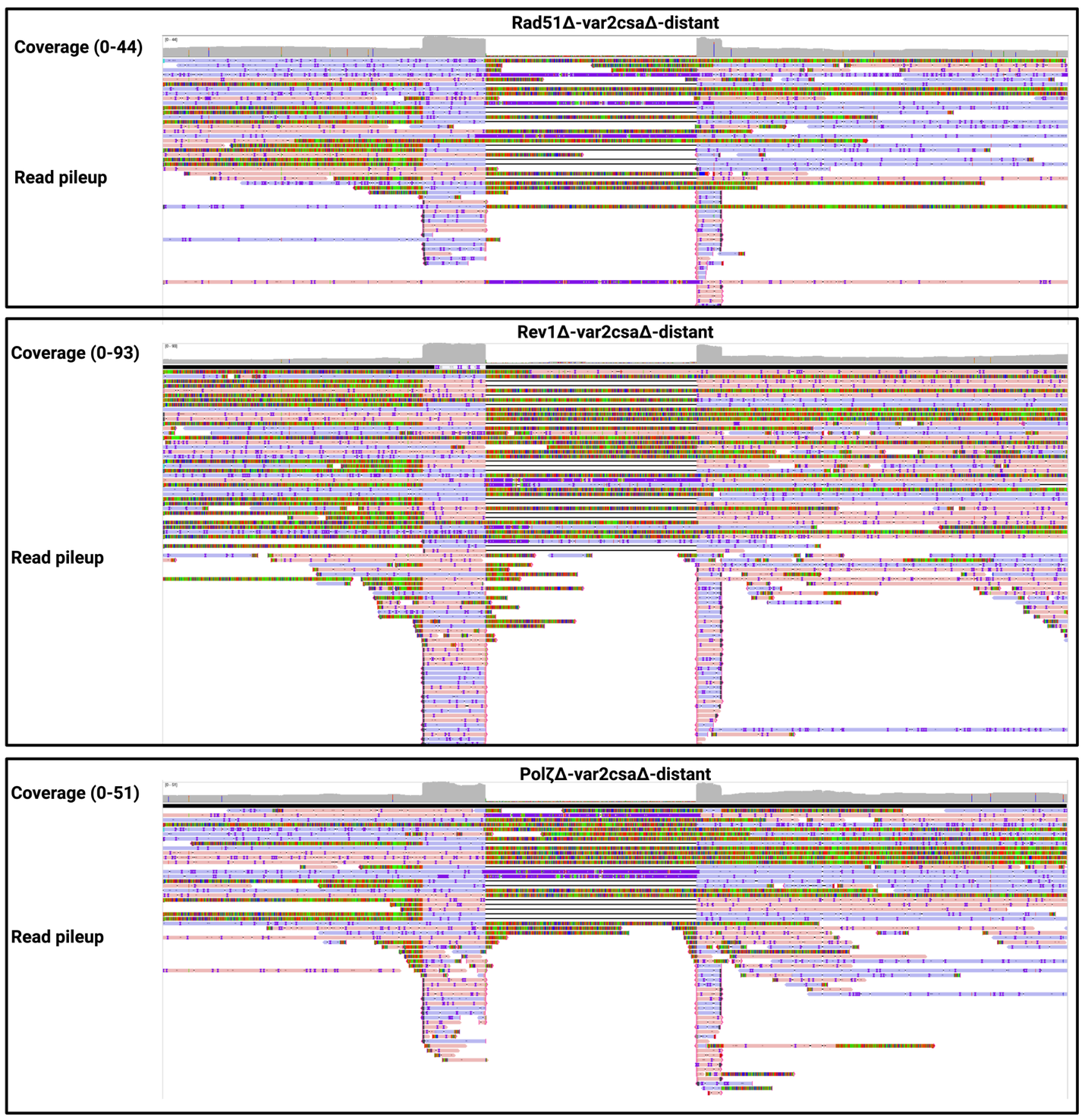


Supplementary Figure 7: Local genome assembly of (A) *PfRad51Δ-var2csaΔ*-distant and (B) *PfPolζΔ-var2csaΔ*-distant: The red blocks represent the homology block1 and the blue box represents homology block 2. The sequence between these two blocks span ~18kb and consists of sequences derived from the donor plasmid in multiple copies (at least 2). The sequence overlap between the chromosomal sequence to homology block to plasmid sequence is most consistent with homologous recombination. The junctions of the recombination sites are shown at the base pair level in the bottom of the figure.

A: *PfRad51Δ-var2csaΔ*-distant


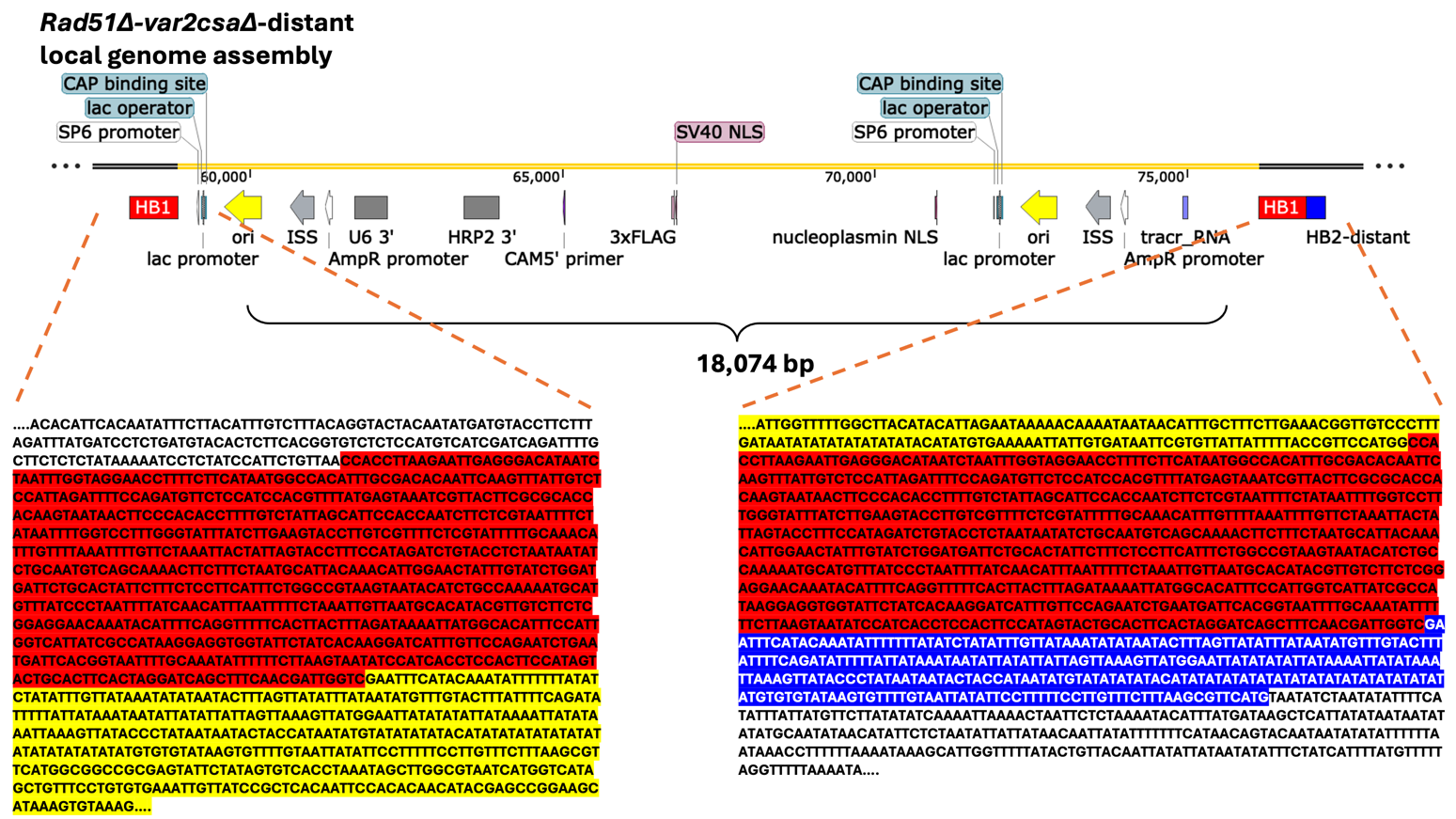


B: *PfPolζΔ-var2csaΔ*-distant


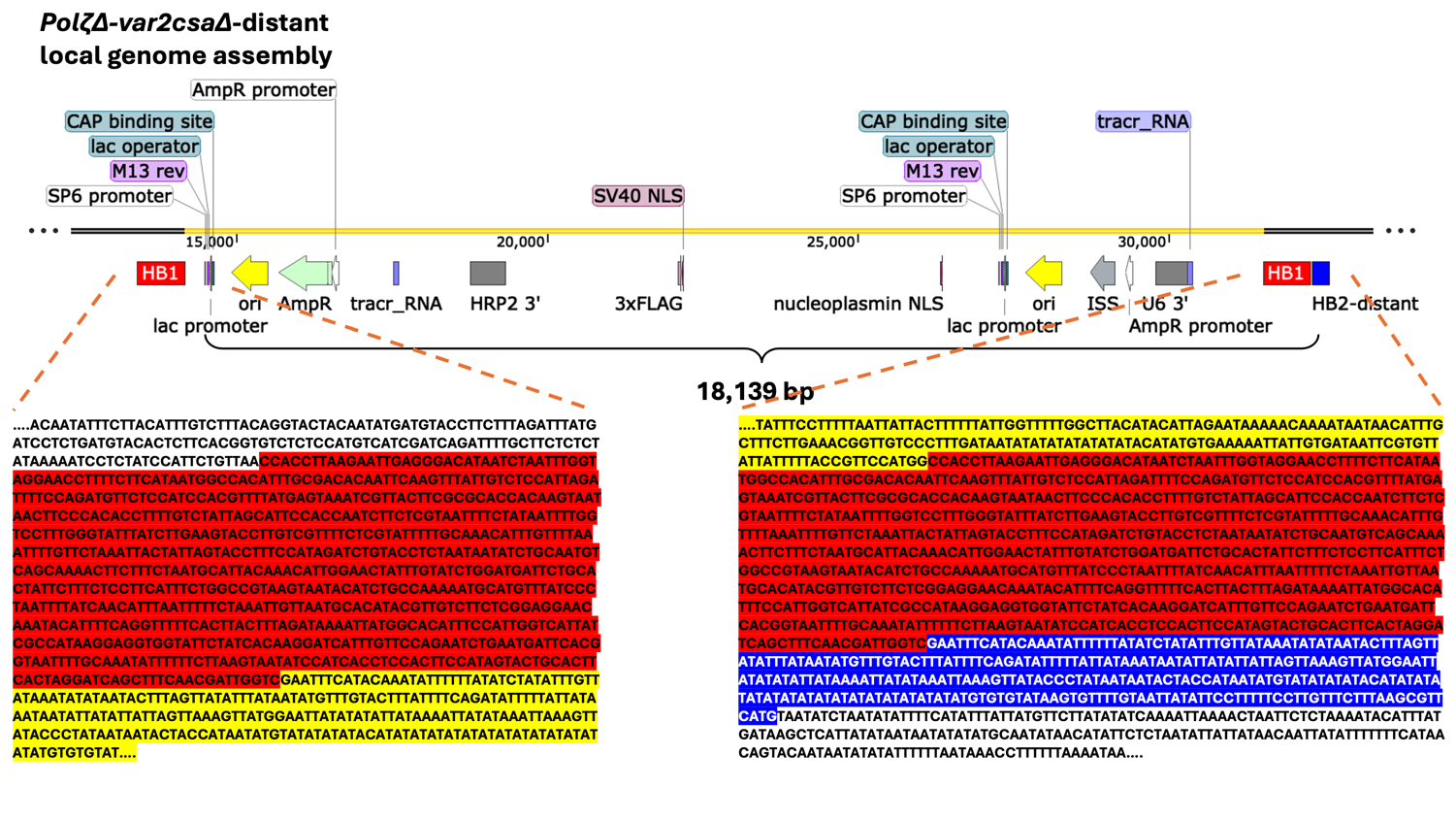

Supplement: gkaf1275_Supplemental_File [file gkaf1275_supplemental_file.docx]
